# Geriatric syndromes extraction from discharge summaries: a new dataset, annotation scheme and initial findings

**DOI:** 10.3389/fdgth.2026.1591050

**Published:** 2026-07-13

**Authors:** Imane Guellil, Salomé Andres, Atul Anand, Bruce Guthrie, Fahrurrozi Rahman, Abul Hasan, Huayu Zhang, Honghan Wu, Beatrice Alex

**Affiliations:** 1 Advanced Care Research Centre - Usher Institute, University of Edinburgh, Edinburgh, United Kingdom; 2 Department of Cancer and Genomic Sciences, University of Birmingham, Birmingham, United Kingdom; 3 Institute of Health Informatics, University College London (UCL), London, United Kingdom; 4 Nuffield Department of Primary Care Health Sciences, Oxford University, Oxford, United Kingdom; 5 School of Health & Wellbeing, University of Glasgow, Glasgow, United Kingdom; 6 School of Mathematical & Computer Sciences, Heriot-Watt University, Edinburgh, United Kingdom

**Keywords:** annotation guidelines, corpus annotation, geriatric syndromes, named entity recognition, natural language processing, NLP

## Abstract

**Introduction:**

Geriatric syndromes (GS), such as falls, dementia, delirium and malnutrition, are complex clinical conditions affecting older adults which involve multiple organ systems and have major impact on quality of life and care. GS cut across disease categories, and are poorly represented in structured electronic health records. Natural language processing (NLP) offers an opportunity to extract valuable GS-related information from unstructured clinical text, such as hospital discharge summaries. However, the lack of high-quality annotated datasets limits the effectiveness of NLP models in this domain. This study introduces a manually annotated corpus designed for GS detection, enabling more accurate identification and classification of GS.

**Methods:**

We developed a comprehensive and detailed annotation scheme to label 12 common GS from hospital discharge summaries, incorporating key attributes such as diagnosis type, negation and event occurrence. The corpus consists of 2,040 manually annotated discharge summaries from National Health Service (NHS) Lothian hospitals in Scotland. To assess the effectiveness of NLP in extracting GS, we experimented with multiple pretrained transformer-based models, including base BERT (general-domain), BioBERT (biomedical-domain), BioClinicalBERT (clinical-domain) and BERT-cased (the cased English BERT checkpoint). The models were fine-tuned and tested for two types of tasks: named entity recognition (NER) and document-level labelling. We also considered an extra task of detecting contextual information with each GS mention (e.g., history, suspected, in-hospital). When context information is considered, two new tasks are called NER-C and DL-C, for NER and document-level labelling with context respectively.

**Results:**

Our evaluation showed that, for the document-level labelling task, BERT-cased achieved the highest F1-score (0.897) and BioClinicalBERT performed best when negation was considered (F1-score: 0.888). For the NER task, BioClinicalBERT and BERT-cased achieved an F1-score of 0.883. Frailty (F1 = 1.0), Falls (F1 = 0.973) and Delirium (F1 = 0.946) are the GS entities with the best performing results. For NER-C, BERT-cased achieved the best F1 of 0.692 and BioBERT performed the worst (F1 = 0.658). In NER-C, the best results were achieved for context-aware falls and frailty labels, particularly when the syndrome was implied rather than explicitly stated. Document-level aggregation helped reduce inconsistencies, but the NER experiments used a flattened CoNLL-compatible representation of the original annotations, in which discontinuous mentions were converted into shortest covering spans and overlapping mentions were merged. Therefore, the reported NER results should be interpreted as baseline performance on a simplified representation of these structures, while low-frequency GS categories and sparse contextual labels also negatively affected model accuracy.

**Discussion:**

This study demonstrates the effectiveness of NLP for extracting geriatric syndromes from unstructured clinical text and introduces a manually annotated corpus with detailed guidelines to support this task. The results also show that model performance is strongly shaped by dataset characteristics. More frequent and lexically clearer syndromes, such as frailty, falls and delirium, achieved the strongest results, whereas rarer categories and low-frequency attribute combinations, such as suspected, referral and some negated or context-specific labels, were harder to learn and yielded lower and less stable scores. Likewise, fine-grained annotation was more challenging than coarse-grained annotation because it increases label sparsity and requires the model to distinguish subtle contextual differences, such as current vs. historical mentions, implicit mentions and in-hospital onset. Entity-level extraction was further affected by discontinuous and overlapping mentions, which are common in clinical narratives and make boundary detection harder, whereas document-level aggregation reduced the impact of these local errors and therefore produced higher scores. These findings underline that data distribution, annotation complexity and mention structure directly influence model performance, and should be central considerations in future work on geriatric syndrome extraction.

## 1 Introduction

Older adults often experience patterns of diseases and syndromes that require a holistic understanding of their care needs. The term “geriatric syndromes” (GS) refers to complex and heterogeneous clinical conditions in older adults that do not fit neatly into discrete disease categories. These syndromes often involve multiple organ systems, posing challenges for both clinical care and research (1, 2). Common GS include falls, frailty, malnutrition, weight loss, delirium, dementia, unspecified cognitive impairment, urinary incontinence, faecal incontinence, pressure injury, visual impairment and hearing impairment.

GS are clinically and policy important because they have major implications for quality of life, independence, discharge planning, long-term care needs and health-service use among older adults. Understanding GS in routinely collected health records can support patient-oriented clinical research, service planning and population-level surveillance of ageing populations. This is particularly important because the care needs of older adults are often shaped not only by individual diagnoses, but also by functional, cognitive, nutritional and social factors that cut across traditional disease categories.

Despite their clinical importance, GS are often poorly represented in coded data because they cut across disease-based classifications (3–5). Instead, valuable information about GS is frequently embedded in the free text of electronic health records (EHRs), such as hospital discharge summaries and general practitioner notes. Extracting insights from these types of unstructured texts to understand the health and social care needs of older populations is therefore an emerging area of research interest (6, 7).

However, GS extraction from clinical free text is linguistically challenging. Mentions may be explicit, such as a documented fall or diagnosis of delirium, but they may also be negated, historical, suspected, referred for further assessment or implied indirectly through descriptions of mobility, cognition, continence or nutrition. Clinical narratives may also contain discontinuous and overlapping entities, where evidence for a syndrome is distributed across non-contiguous spans or where the same textual span supports more than one GS label. These features make GS extraction more complex than simple keyword matching or coarse document-level classification.

NLP remains underutilised in patient-oriented clinical research. While state-of-the-art NLP and machine learning (ML) models have facilitated GS extraction, their performance is highly dependent on the availability of high-quality annotated data, which are essential for training robust models. Existing NLP benchmarking efforts primarily use publicly available datasets and are very task- and performance-oriented, and often do not place older patient needs at the core of the research. Consequently, there is a disconnect between the capabilities of clinical NLP methods and the requirements of medical professionals interacting directly with an ageing population (8). Additionally, several challenges remain, including limited generalisability across datasets, handling discontinuous and overlapping entities and ensuring annotation consistency. Furthermore, much of the previous research in this space has focused on social media and biomedical literature rather than clinical records, limiting its direct relevance to real-world patient care.

**Figure 1:**
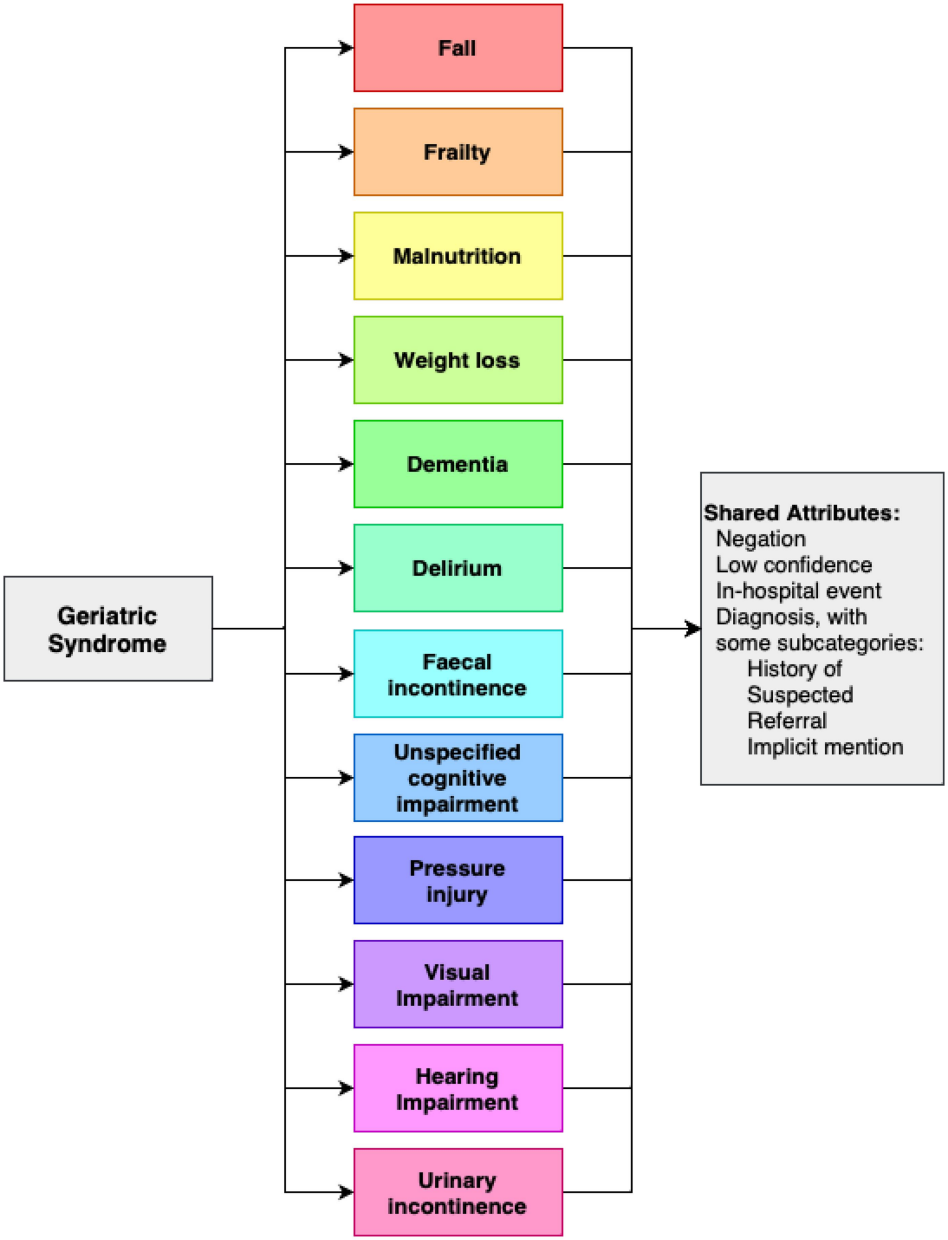
Overview of the geriatric syndrome (GS) annotation schema used in this study. The figure shows the 12 GS entity categories and the contextual attributes applied to annotated mentions, including negation, low confidence, diagnosis status and in-hospital event. These attributes allow the corpus to distinguish simple GS mentions from more clinically specific cases, such as historical, suspected, referred, implicit or hospital-acquired events.

To address these challenges, we present a manually annotated corpus of clinical free-text data, where GS are systematically identified, labelled and categorised using a rigorous annotation scheme. We focus on 12 GS frequently observed in older patients, such as falls, malnutrition, dementia, delirium, urinary incontinence and faecal incontinence (9–11). Each GS is associated with contextual attributes, including Diagnosis, which is categorised into *history_of*, *suspected*, *referral* and *implicit_mention*, as well as negation, low confidence and in-hospital event. The complete set of entities and attributes is illustrated in Figure 1. Our methodology involves constructing a high-quality annotated corpus^1^ and evaluating multiple embedding models trained on this data, primarily transformer-based models such as BioBERT and BERT variants, across different levels of annotation granularity.

### 1.1 Contributions

Despite significant progress in NLP-based detection of geriatric syndromes, several challenges remain. Existing work is limited by the lack of standardised datasets, limited availability of annotation guidelines, inconsistent evaluation settings and relatively little attention to clinically meaningful contextual attributes. To address these gaps, this paper makes the following contributions:

1. **A clinically informed annotation scheme for geriatric syndromes.** We develop a detailed annotation scheme for 12 GS commonly affecting older adults, including Falls, Frailty, Malnutrition, Weight loss, Delirium, Dementia, Unspecified cognitive impairment, Urinary incontinence, Faecal incontinence, Pressure injury, Visual impairment and Hearing impairment. The scheme was developed in collaboration with clinicians and is designed to reflect clinically relevant distinctions in discharge summaries.
2. **A manually annotated corpus of discharge summaries.** We create a manually annotated corpus of 2,040 de-identified NHS Lothian discharge summaries provided through DataLoch,^2^ focusing on older patients. This corpus provides entity-level annotations that can support both information extraction and document-level labelling tasks. The dataset is available upon successful application to DataLoch, subject to information-governance approval.
3. **Context-rich annotations beyond coarse GS detection.** In addition to the 12 core GS categories, the annotation scheme captures clinically relevant contextual attributes, including negation, low confidence, history of, suspected, referral, implicit mention and in-hospital event. The original BRAT annotations also allow discontinuous and overlapping mentions, reflecting the fact that clinical evidence for a syndrome may be distributed across non-contiguous tokens or may support more than one syndrome label. However, the baseline modelling experiments reported in this paper do not directly model these structures: discontinuous mentions are converted into shortest covering spans and overlapping mentions are merged during CoNLL conversion.
4. **Initial baseline experiments for GS extraction.** We evaluate several embedding-based sequence-labelling models, including GloVe, BERT-uncased, BERT-cased, BioBERT and BioClinicalBERT, for entity-level GS extraction and document-level GS labelling under fine-grained, coarse-grained and coarse-grained-with-negation settings. These experiments are intended as initial baselines demonstrating the usability of the annotated corpus, rather than as fully optimised model comparisons.
5. **Annotation of discontinuous and overlapping geriatric syndrome mentions.** The annotation scheme explicitly supports discontinuous and overlapping entities in the original BRAT annotations. This is important for clinical free text, where evidence for a geriatric syndrome may be distributed across non-contiguous tokens, as in cases where two separated words jointly express one clinical concept, or where the same textual span supports more than one syndrome label. These annotations preserve clinically meaningful mention structures that are often lost in standard sequence-labelling formats.
  However, the baseline modelling experiments in this paper use the CoNLL format, which requires a flat token-level representation. Therefore, for the experiments reported here, discontinuous mentions were converted into their shortest contiguous covering spans and overlapping mentions were merged during preprocessing, following the flattening strategy described by Stanovsky et al. (12) and Dai et al. (13). As a result, the current experiments should be interpreted as baseline results on a CoNLL-compatible representation, rather than as direct modelling of discontinuous or overlapping entities. The original BRAT annotations nevertheless retain these structures and provide a useful resource for future work on span-based, hypergraph-based or transition-based models designed to handle discontinuous and overlapping clinical entities directly.

Together, these contributions position the paper primarily as a dataset and annotation-resource contribution for GS extraction from clinical free text, with baseline modelling results to support future methodological development.

## 2 Related work

Recent research on the detection of GS from electronic health records (EHRs) has gained increasing attention due to its potential to improve early diagnosis, support clinical decision-making and facilitate population-level surveillance. Using NLP techniques, previous studies have employed various approaches, including classifying GS presence at the sentence, document or patient level, extracting syndrome-related characteristics and profiling cohorts. While advanced methodologies, such as machine learning (ML) and deep learning (DL), are being increasingly adopted, classical rule-based methods remain widely utilised due to their interpretability and methodological simplicity.

Studies have employed two broad task types: classification and information extraction. Classification work has primarily targeted binary detection of GS presence (14–23), with some studies extending to multiclass settings to distinguish frailty categories (20) or delirium likelihood (17), and others predicting future onset of falls (24, 25). Information extraction studies have focused on identifying symptom characteristics and profiling patient cohorts across a range of GS including urinary incontinence, falls and malnutrition (26–29), with several integrating classification and extraction to support broader phenotyping tasks such as dementia (30, 31), malnutrition (32) and mild cognitive impairment (33).

Most datasets are derived from institution-specific EHRs and are therefore not publicly accessible, with the exception of one dataset (15). They cover a variety of document types, including progress notes (14, 15, 31, 32), primary care notes (24) and visit and coordination notes (28), and originate from a range of institutional sources including hospitals, care facilities and large-scale data repositories. Differences in clinical documentation practices, terminologies and population demographics present challenges for generalisability and comparability, while institutional policies and ethical considerations further constrain data sharing and external validation. Table 1 situates our corpus within this broader landscape, contrasting general-purpose resources with task-specific, institution-bound datasets across note type, scale, annotation targets and accessibility. Our contribution fills a gap by providing GS-focused discharge summary annotations with richer contextual labels than most existing benchmarks.

**Table 1:** Comparison of general-purpose, clinical-text resources and prior datasets/studies relevant to geriatric syndromes (GS), alongside our GS discharge-summary corpus.

Dataset/study	Source + note type(s)	Size (reported)	Labels/task + key contribution
MIMIC-III (34)	BIDMC ICU EHR + free-text notes	38,597 patients; 49,785 admissions; 53,423 ICU stays	General-purpose de-identified ICU EHR widely used for phenotyping/NLP; not a GS-specific gold annotation set.
MIMIC-IV (35)	BIDMC EHR + de-identified notes (versioned release)	364,627 patients, 546,028 hospitalisations and 94,458 ICU stays	Contemporary general-purpose EHR/notes resource; useful substrate but not GS-focused gold annotations.
i2b2 2010 (concept/assertion/relation) (36)	De-identified clinical notes (incl. discharge summaries)	426 docs (170 train/256 test)	Gold annotations for clinical concepts + assertions/relations; task-specific (not GS-targeted).
n2c2 2018 Track 2 (ADE/medication) (37)	Discharge summaries (selected from MIMIC-III)	505 discharge summaries (303 train/202 test)	Gold annotations for meds, attributes, reasons, ADEs + relations; ADE-focused (not GS-targeted).
Scharp et al. (28)	Home healthcare clinical notes	39,179 episodes; 1,098,419 notes; 29,981 pts	NLP to identify urinary incontinence symptom concepts (symptom extraction + note-level classification).
Cheligeer et al. (14)	3 acute-care hospitals (progress notes)	4,323 adult inpatients	BERT-based inpatient fall detection (binary classification).
Dormosh et al. (24)	Primary care GP notes	35,357 individuals; 4,734 fallers	Topic features from notes to predict future falls (risk prediction).
Mishra et al. (25)	Senior living facility (nursing notes + meds)	NR${}^{a}$	Fall risk prediction using free-text nursing notes + medications.
Maclagan et al. (31)	Primary care EMR consult/progress notes	526 dementia cases; 44,148 controls	Dementia identification from primary care notes (patient-level classification).
Shao et al. (22)	VA EHR notes + structured data	˜1M notes; training 5K/5K per race; chart review 1.2K	ML risk scores for probable undiagnosed dementia; race-stratified models.
Alkhalaf et al. (32)	Residential aged care (progress notes)	4,405 clients; 1,469 malnourished	NLP extraction of malnutrition factors; highlights undercapture in structured fields.
Penfold et al. (33)	Clinical notes (integrated delivery system)	NR${}^{a}$	ML model to predict MCI using NLP signals when screening absent.
Fu et al. (falls) (18)	EHR clinical notes	NR${}^{a}$	Context-aware fall identification with hybrid BERT + rules; evaluated at sentence/document/patient levels.
Wang et al. (delirium) (23)	Multi-hospital admissions + imaging report text	3,862 admissions; 994 delirium-labeled	Delirium identification improved via sentiment-based NLP features.
Ge et al. (delirium) (19)	Notes from 9 hospitals	>10,000 pts; 1.5M notes; 200,471 sentences labeled	Sentence-level delirium detection with expert-labeled sentences (transformers best).
Nakatani et al. (38)	Japanese EMR nursing records	335 fallers; 408 non-fallers	Fall prediction using NLP of nursing records (case-control).
Lorenzoni et al. (27)	Falls surveillance narratives + pre-coded fields	202 fall records	Text mining + clustering to characterise fall patterns.
Martin et al. (frailty aspects) (20)	Encounter notes (sentence annotation)	326 patients; 155,952 sentences	Sentence-level labels for 4 actionable frailty aspects; predictive modeling.
Soysal et al. (weight loss in dementia) (29)	Dementia care EHR (clinical notes)	11,607 dementia patients	NLP-derived weight-loss mentions linked to mortality/hospitalisation.
Du et al. (cognitive decline) (16)	Mass General Brigham clinical notes	Dev: 4,949 sections/1,969 pts; Test: 1,996 sections/1,161 patients	LLM prompting vs traditional models; ensemble improves performance.
This work (GS discharge summaries)	NHS Lothian discharge summaries	2,040 discharge summaries (manually annotated)	GS-focused corpus + guidelines for 12 GS with context (negation, uncertainty, diagnosis status, in-hospital onset) enabling entity-level + document-level supervision, including overlapping/discontinuous mentions.

${}^{a}$NR, not reported

To establish high-quality reference standards, most datasets are manually annotated at sentence, document or patient level. Inter-annotator agreement (IAA) is commonly reported using variants of Kappa (14, 16, 22), F1-score (17, 18) and pairwise agreement (19), though inconsistencies in methodology across studies make replication challenging. Despite the need for standardisation, only two studies have explicitly reported annotation guidelines (17, 20), and our work addresses this gap directly by providing fully documented annotation guidelines.

Despite advances in ML and DL, rule-based methods remain prevalent. The majority of studies utilise these techniques in isolation, with only a few integrating DL and rule-based methods into hybrid frameworks (18) or combining LLMs with ML ensembles (16). The continued use of rule-based methods, including regular expressions, is primarily attributed to their simplicity and interpretability (14, 17, 32), though their scalability poses significant challenges on large or heterogeneous datasets.

Beyond rule-based approaches, traditional ML techniques such as Support Vector Machines, Logistic Regression and Random Forest remain popular due to their relative simplicity and interpretability, with some studies also employing domain-specific tools such as NimbleMiner (28) and GATE (29). More recently, fine-tuned Transformer-based models (18, 19) and prompt-based learning with LLMs such as GPT-4 and Llama 2 (16) have been adopted, offering improved generalisability but introducing challenges around computational cost, domain adaptation and explainability. This is particularly relevant in clinical settings, where data governance constraints preclude the use of proprietary models that require sending text to third-party servers, a consideration that applies with added force in trusted research environments, where access to LLMs for unconsented data is not currently approved.

Input representations range from classical features such as TF-IDF and n-grams (23) to distributed word embeddings such as Word2Vec and GloVe (15, 18, 20, 25, 28), with more recent work adopting Transformer-based models such as BioClinicalBERT and RoBERTa to better capture domain-specific linguistic nuances (14, 18–20). Topic modelling approaches such as LDA (22, 27) and Top2Vec (24) have also been explored to uncover thematic patterns in EHRs, though they remain underutilised relative to embedding-based methods, partly due to challenges in evaluation and interpretability. In this work we evaluate both GloVe embeddings and Transformer-based models, reflecting the range of approaches documented in the literature.

For a comparison of our contribution to prior work, please refer to Table A1 in the Supplementary Appendix.

## 3 Annotation guidelines

There is no consensus on an exhaustive list of GS. In the context of this study, we reviewed the current literature and organised a Public Participation Involvement and Engagement (PPIE) workshop for older adults to propose a patient-oriented NLP tool.^3^ Based on the information collected, we collaborated with an expert panel of clinicians using a consensus approach to select 12 GSs (detailed in Section 3.1). We selected these GSs because they are common and have a significant impact on patients’ quality of life, often requiring substantial medical intervention.

### 3.1 Annotation labels

In the context of this work, each type of GS represents a label. The following list provides an example for each of the different types:

1. **Fall:** Three falls in last yr
2. **Frailty:** Increasing frailty but manages well, mostly independent but gets help with heavy shopping
3. **Malnutrition:** Patient appears cachectic
4. **Weight loss:** She reports some concerning weight loss over the last 3 months
5. **Delirium:** On admission, she was confused. This settled to some extent with pain relief and rehydration.
6. **Dementia:** Diagnosed with Alzheimers about 18 mths ago
7. **Unspecified cognitive impairment:** Short term memory loss secondary to stroke 3 years ago
8. **Urinary incontinence:** Urgency, can’t get to toilet in time
9. **Faecal incontinence:** He has been struggling with faecal leakage for a week
10. **Pressure injury:** Noted decubitus ulcer on the right heel
11. **Visual impairment:** Registered blind, since he was 55
12. **Hearing impairment:** Very deaf

### 3.2 Annotation attributes

Annotation attributes are additional features or characteristics associated with the tag. We use four main attributes: Negation, Low confidence, Diagnosis and In-hospital event.

#### 3.2.1 Negation

Negation can be formulated explicitly with “no” or “not” but can also appear as acronyms (e.g., NAD, meaning nothing abnormal discovered) or be inferred from the text. The annotator did when possible, not include the negation word(s) in the selection of text but simply highlight the words which reference the AE and add the negation attribute to the label. The following examples highlight how negation can be captured:

- “She slipped but did not fall.”—This should be annotated *Falls* with the negation attribute.
- “Patient can hear me well and does not require hearing aids.”—This should be labelled *Hearing impairment* with the negation attribute.
- “His daughter says that he has a good appetite and has been maintaining weight in the last year.”—From this, we can infer the patient has not lost weight and the label *Weight loss* can be used with the negation attribute.

#### 3.2.2 Low confidence

Another attribute associated with GS is *Low confidence*. This attribute refers to the annotator’s confidence in assigning a given GS label to the highlighted text span, rather than uncertainty in the patient’s underlying clinical diagnosis itself. It should be selected when the annotator judges that the textual evidence linking the span to the GS is weak, ambiguous or unusual. There are two main situations in which we recommend the use of this attribute:

- If there is insufficient information in the document
- If the annotator is faced with an unusual description of events which is, according to the writer of the letter, to be associated with a GS.

The purpose of the *Low confidence* attribute is to preserve potentially relevant but uncertain examples while marking them as weaker evidence. This provides nuance in the final annotated document and distinguishes straightforward mentions from those requiring more interpretive judgement. For example:

- “The patient was incontinent of urine on the ward”—This is an element of text which should be annotated as *Urinary incontinence* and the *Low confidence* attribute should not be selected in this context.
- “She was admitted with delirium, was confused in the ambulance and had visual hallucinations at home. Her daughter said she was describing children running around but they were by themselves in the house. The delirium resolved with rehydration.”—Hallucinations are rarely present in delirium but it seems to be described by the author of the letter as such. It should therefore be annotated with the label *Delirium* and the *Low Confidence* attribute.

#### 3.2.3 Diagnosis

This attribute allows the annotator to further qualify the annotated text when the GS is not directly present in the patient’s admission, attendance or report. For this purpose, four tags are available:

- *History of*, when selected, qualifies GS which has happened in the past. This can be mentioned in the patient’s past medical history or something which happened before the admission or attendance and is not related to the current clinical presentation. For example: “Past medical history includes longstanding urinary incontinence.”—The GS *Urinary incontinence* should be tagged with the attribute *History_of*, because it refers to a pre-existing condition documented as background history rather than the main current presentation. The *History_of* attribute is intended for pre-existing or past conditions mentioned as background history, and not for syndromes that are clearly presented as part of the patient’s current admission, attendance or active clinical problem.
- *Suspected*, qualifies when the writer of the clinical document suspects the patient has a GS (note this is not the suspicion of the annotator—if the annotator is unsure about a GS to be annotated, nuance can be added with the attribute *Low confidence* instead (see previous paragraph)). This attribute can also be used in annotating screening tests which require further investigations to confirm a diagnosis. For example: “The Old Age Psychiatry team suspects this might be dementia, CT scan is pending.”—The GS *Dementia* should be tagged with the attribute *Suspected*.
- *Referral* qualifies when a patient is being referred to a different healthcare professional for the diagnosis of their GS. For example: “She was discharged home with an increased package of care and a referral to the dementia clinic has been made.”—The GS *Dementia* should be tagged with the attribute *Referral*.
- *Implicit mention* qualifies a GS which is mentioned in the text but does not apply to the patient. This includes descriptions of the patient’s family history or diagnosis given to their spouse. It also should be used when the text details hypothetical situations (“if” statements) or risks of a GS happening (e.g., risk of falls). “The community occupational therapy team will review the patient’s house after his discharge to evaluate and minimise the risk of falls.”—This is a hypothetical mention of the GS *Falls* (with “risk of”). It should therefore be annotated as *Falls* with the attribute *Implicit mention*. Finally, if the *Diagnosis* field is left empty, this signifies that the GS has been diagnosed, is present during the admission or attendance or that it is the reason for the admission or attendance. For example: “He was admitted with a fall and developed delirium during his admission due to a urinary tract infection.”—The two of these diagnoses have happened in direct relation to the admission and therefore should be annotated without any additional *Diagnosis* attribute.

#### 3.2.4 In-hospital event

This attribute allows the annotator to identify GS for which the onset took place in hospital. For example, if a patient has a fall on the ward after surgery or if they were admitted with no symptom of delirium but developed acute confusion during their stay.

This attribute aims to capture the location of the event rather than the location of the diagnosis. This is important to keep in mind since some GS are diagnosed during the admission, but their onset will have taken place before the patient came to the hospital. For example, if a patient had a fall two days prior and is admitted and treated for an infection. On day 3, they reported pain on their wrist and the x-ray of their arm showed a fracture but clearly this *Fall* happened at home 5 days ago, therefore the attribute *In-hospital event* should not be selected.

### 3.3 Discontinuous and overlapping entities

#### 3.3.1 Discontinuous entities

Medical texts often contain distant entities which need to be annotated together to make sense of the label which is being recognised. Discontinuous entities, also named distant entities or fragmented entities, are often used for this purpose (39). For example:

- “Her memory had apparently been progressively deteriorating over several months”—These two distant tokens should be annotated as one discontinuous entity with the label *Unspecified cognitive impairment*

When deciding to extract discontinuous entities, the annotator should aim to select terms which are as close as possible to each other in the text while retaining the meaning of the label, as shown in the examples below:

- item “Was started on supplements after dietitian review”—Note that if a patient takes supplements following a dietitian’s review, we can assume they are malnourished. Hence, *supplements* and *dietitian* should be annotated as two discontinuous entities
- “Carers report he lost 2 to 3 stones over 6 months”—This is a discontinuous entity.

The use of discontinuous entities should not be used when annotating two different, isolated symptoms which are associated with the same GS. For example:

- “She was confused on the ward”—Is an appropriate token to select for the label *Delirium*
- “She was agitated on the ward”—Is also an appropriate token to select for the label *Delirium*
- “She was confused and agitated on the ward”—since both of these symptoms, in isolation, are sufficient to detect delirium, the tokens highlighted in this example must be annotated as two separate entities with the label *Delirium*. The discontinuous entity tool should not be used in this case.

#### 3.3.2 Overlapping entities

Medical texts also tend to contain overlapping entities which require the annotator to highlight the same element of text twice and this can be used in combination with the distant entities (39). For example:

- “He is doubly incontinent”—This can be annotated as the overlap of *Urinary incontinence* and *Faecal incontinence* and does not require the use of distant entities.

## 4 Data annotation

**Figure 2:**
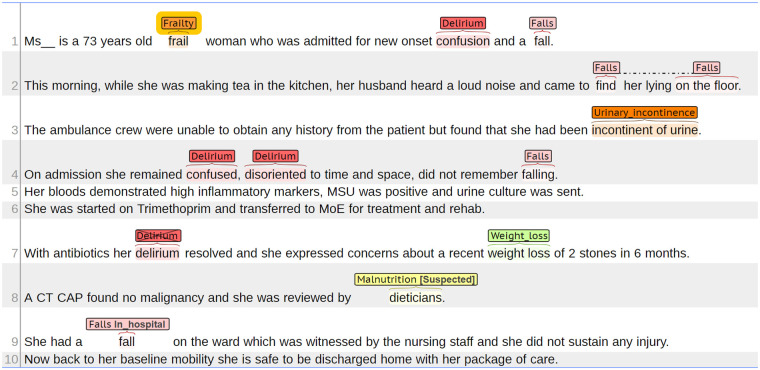
A synthetic example annotated in BRAT, showing different entities and attributes.

We used BRAT,^4^ a web-based text annotation tool (40), to create the annotations. Figure 2 presents a typical example, showcasing some of the annotation labels and attributes defined in our annotation guidelines. As we are unable to share real patient data due to their sensitive nature, this example is synthetic.

### 4.1 Dataset

We annotated data for a cohort of 200 patients, born on or before 1949, from NHS Lothian hospitals. 100 individuals are aged between 70 and 79 years and 100 are aged 80 years or over. All patients had to have had more than two recent hospital visits. 100 individuals had at least one hospitalisation coded for delirium (30 individuals), falls or fainting (20 individuals) and injurious falls (50 individuals). For this cohort, DataLoch provided us with a set of de-identified discharge summaries (2,040) belonging to these patients. Annotations were performed by two clinicians (both co-authors): Annotator1, a junior doctor with several years of clinical experience, and Annotator2, a geriatrician.

Table 2 presents a detailed breakdown of sentence- and token-level statistics across the training, development and test sets. The complete dataset comprises 64,336 sentences across 2,040 discharge summaries, of which 3,215 sentences (5.0%) contain at least one geriatric syndrome mention. The overall token density of 0.047 indicates that approximately 4.7% of all tokens in the corpus are annotated as part of a geriatric syndrome entity, while the mean sentence-level token density of 0.147 shows that when a sentence does contain annotations, an average of 14.7% of its tokens are labelled. The high standard deviation in sentence density (0.185) reflects the heterogeneous nature of clinical documentation, where geriatric syndromes are mentioned sporadically throughout discharge summaries rather than uniformly distributed. The majority of sentences (61,121; 95.0%) contain no annotations, which is not unexpected given the nature of discharge summaries. These statistics demonstrate that while GS mentions are relatively sparse at the corpus level, they are substantive when present. The annotation workload primarily involved identifying and precisely labelling these 3,215 informative sentences and their 6,065 entity tokens across fine-grained categories with multiple attributes.

**Table 2:** Corpus statistics for the NHS Lothian dataset, including number of sentences, labelled sentences, unlabelled sentences, labelled tokens and tokens, as well as token density and mean/median/standard deviation (std) sentence density.

Statistics	Train	Dev	Text	Overall
Sentences	41,256	10,338	12,742	64,336
Labelled sentences	2,070	512	633	3,215
Unlabelled sentences	39,186	9,826	12,109	61,121
Labelled tokens	3,941	979	1,145	6,065
Tokens	84,496	19,667	24,753	128,516
Token density	0.047	0.050	0.047	0.047
Mean sentence density	0.144	0.144	0.160	0.147
Median sentence density	0.079	0.091	0.077	0.083
Std sentence density	0.181	0.174	0.208	0.185

Sentence density is calculated as as the number of tagged tokens divided by the total number of tokens per sentence.

From the 2,040 discharge summaries, 50 documents (24,872 words) were used as a pilot dataset. In this phase, both annotators independently annotated the same set of documents, after which they held a consensus meeting to review and resolve all inconsistencies. This adjudication process helped identify ambiguous cases and led to refinement of the annotation guidelines through the addition of clarifications and further examples. The remaining 1,990 documents were then annotated for training and validating the model using the revised guidelines established during the pilot phase. Among them, 409 documents (190,505 words) were randomly selected as the gold-standard test set and, as in the pilot phase, were independently annotated by the same two annotators. All disagreements in this test subset were subsequently discussed and resolved, yielding a final adjudicated gold-standard version based on annotator agreement. The remaining 1,581 documents, used for model training (1,265 documents/612,338 words) and validation (319 documents/151,981 words), were annotated by a single annotator (Annotator1). This approach is standard practice in clinical NLP and aligns with the methodology of (41), where one annotator was responsible for annotating the entire corpus, while a second annotator was involved only in annotating a subset to establish the gold standard used for determining inter-annotator agreement and final model evaluation.

**Figure 3:**
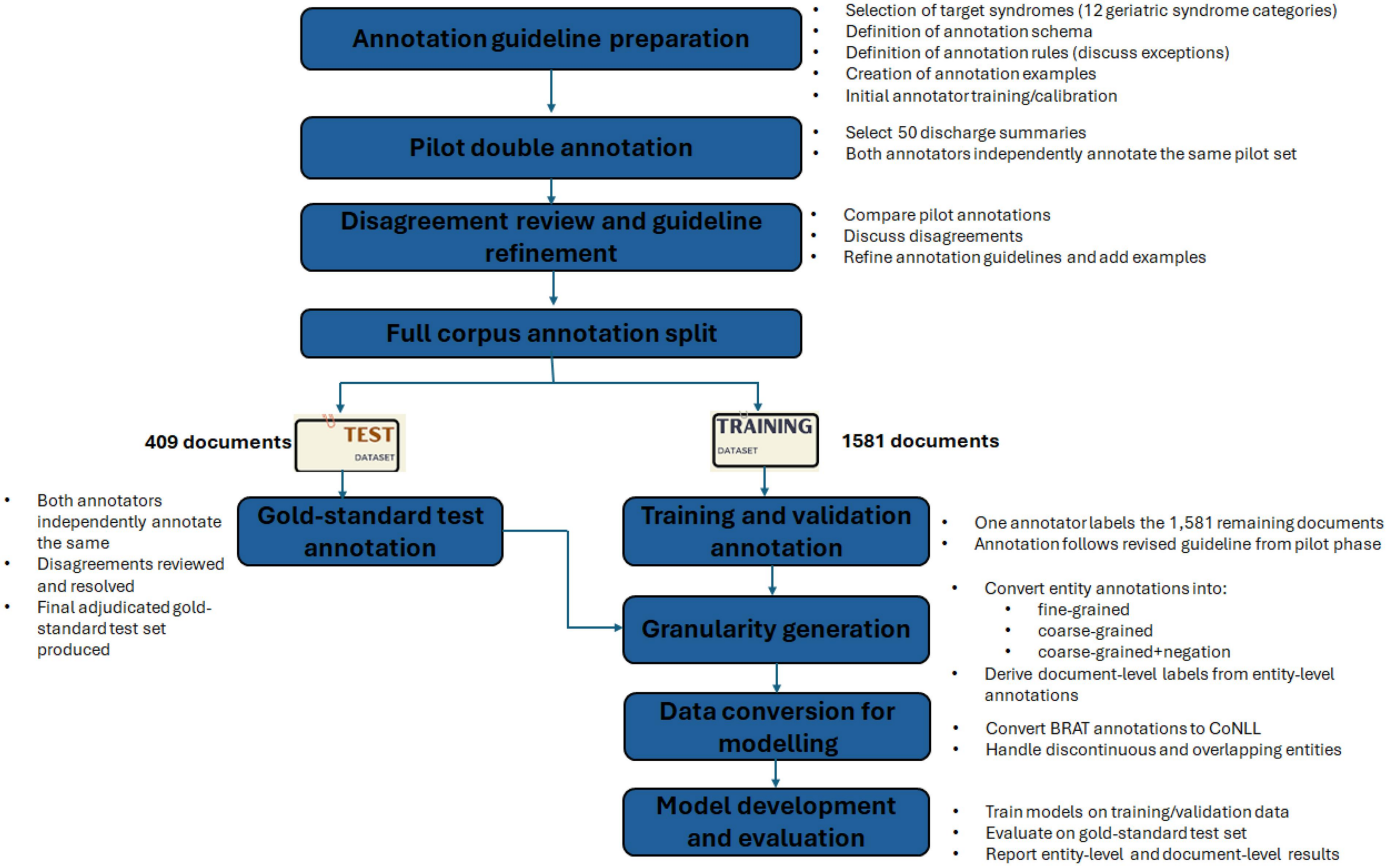
Overview of the dataset development and evaluation workflow.

To improve the clarity and reproducibility of the methodology, Figure 3 summarises the overall workflow used in this study. The figure shows the main stages of dataset development and evaluation, beginning with annotation guideline preparation, followed by pilot double annotation, disagreement review and guideline refinement, corpus splitting, gold-standard test annotation, training and validation annotation, granularity generation, data conversion for modelling and final model development and evaluation. This workflow highlights the distinction between the doubly annotated subsets used for calibration and gold-standard evaluation and the singly annotated subset used for model training and validation.

### 4.2 Annotation levels and granularity

In this study, we focus on both entity- and document-level annotations. The entity-level annotations provide the linguistic evidence for labelling a record with a certain category. This would be useful in an assisted automatic coding scenario, where coders are presented with an automatically coded version of a record and the entity-level annotations provides the evidences for why a certain code was assigned. The document-level annotations are useful to label each record with certain categories. This is mimicking the final output of manual coders when they assign codes to EHR records. It can also feed into prediction algorithms where the linguistic evidence for why a certain record is labelled in a certain way is irrelevant. At the entity level, the goal is to identify each tag along with its position (from the example shown in Figure 2, Span[6:] *frail* → *Frailty*, Span[14:15] *confusion* → *Delirium*). Unlike entity-level annotation, document-level annotation does not consider entity positions or repetitions. For this example, the document-level entities include: *Frailty, Delirium*. In our annotation effort, document-level annotations are inferred automatically from entity-level ones: a document receives a label if at least one entity with that label appears in the document.

The annotation schema also allowed us to consider different levels of granularity: fine-grained, coarse-grained and coarse-grained with negation. For fine-grained annotation, we retain all tags and attributes, resulting in labels such as *Falls* + *history_of* + *In_hospital_events* or *Delirium* + *negation*. This level of detail allows us to capture specific information, such as a patient experiencing *falls*, having a history of *falls* and *falls* occurring in the hospital. In the coarse-grained annotation, we retain only the 12 core geriatric syndrome categories and disregard all additional contextual attributes. These core entities are: *Falls, Frailty, Malnutrition, Weight loss, Delirium, Dementia, Unspecified cognitive impairment, Urinary incontinence, Faecal incontinence, Pressure injury, Visual impairment and Hearing impairment*. For example, in this case, only Falls and Delirium are considered. The coarse-grained setting is useful not only as a simpler benchmark, but also as a clinically and methodologically relevant intermediate task. Methodologically, it allows us to evaluate the core difficulty of detecting geriatric syndrome mentions separately from the added challenge of modelling contextual attributes such as negation, history, referral or suspicion. Practically, coarse-grained detection may be useful in screening, document retrieval or assisted review settings, where the aim is to flag records that mention a syndrome-related concept for subsequent human assessment or downstream contextual processing.

**Figure 4:**
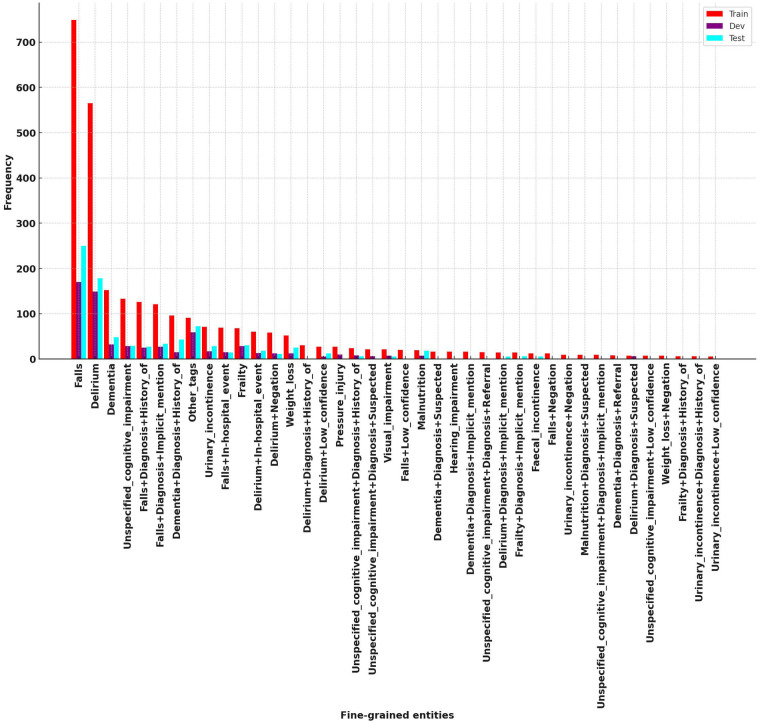
Distribution of fine-grained GS entity labels across the training, development and test sets. Fine-grained labels retain both the core GS category and contextual attributes, such as negation, history, suspected status, referral, implicit mention and in-hospital event. The distribution shows substantial label imbalance, with some clinically frequent or explicit categories occurring more often, while rarer contextual labels have much lower support.

**Figure 5:**
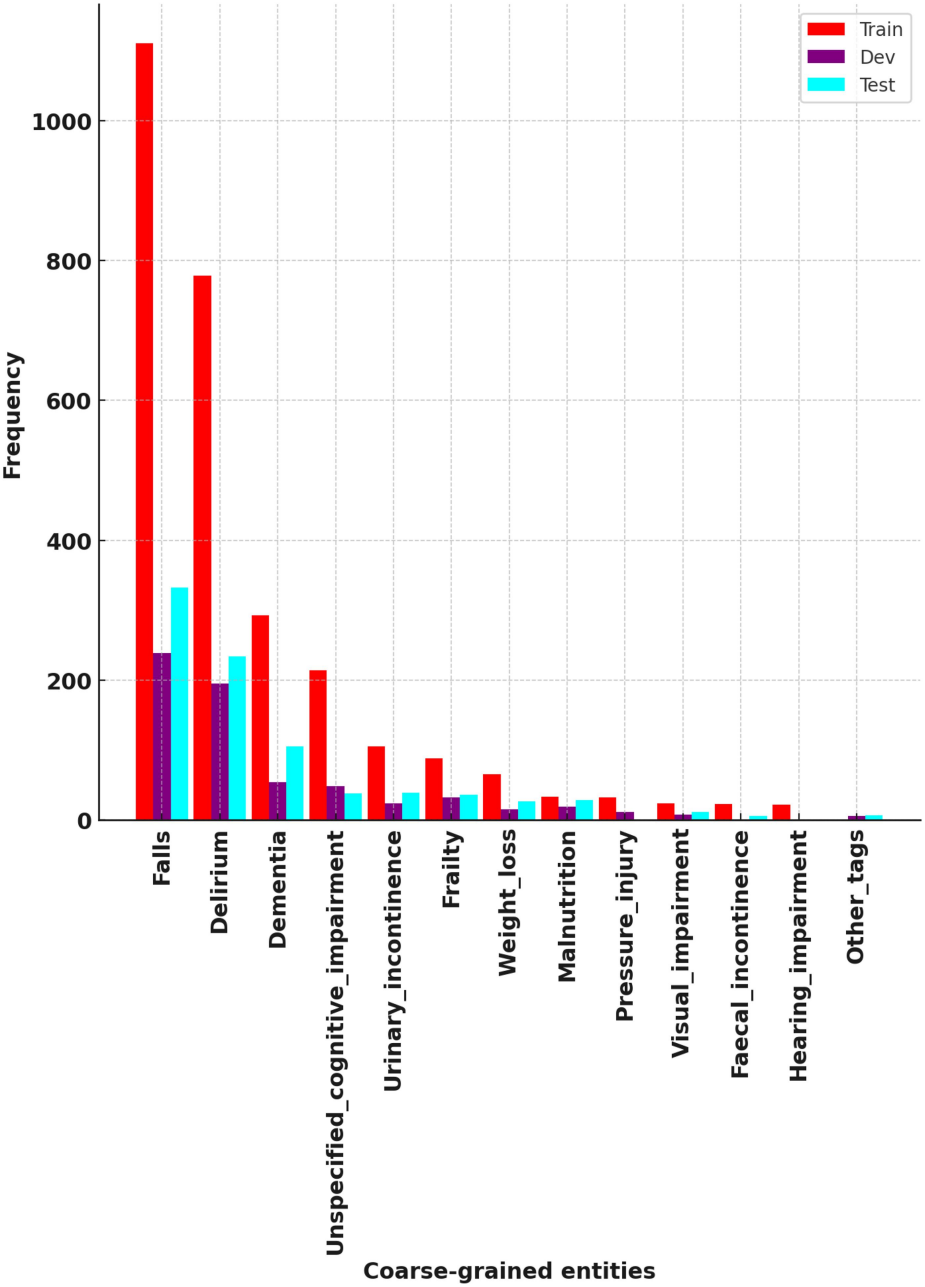
Distribution of coarse-grained GS entity labels across the training, development and test sets. In the coarse-grained setting, contextual attributes are collapsed and only the 12 core GS categories are retained. This view shows the overall frequency of each syndrome category and highlights the uneven distribution of GS mentions across labels, which affects both annotation consistency and model performance.

**Figure 6:**
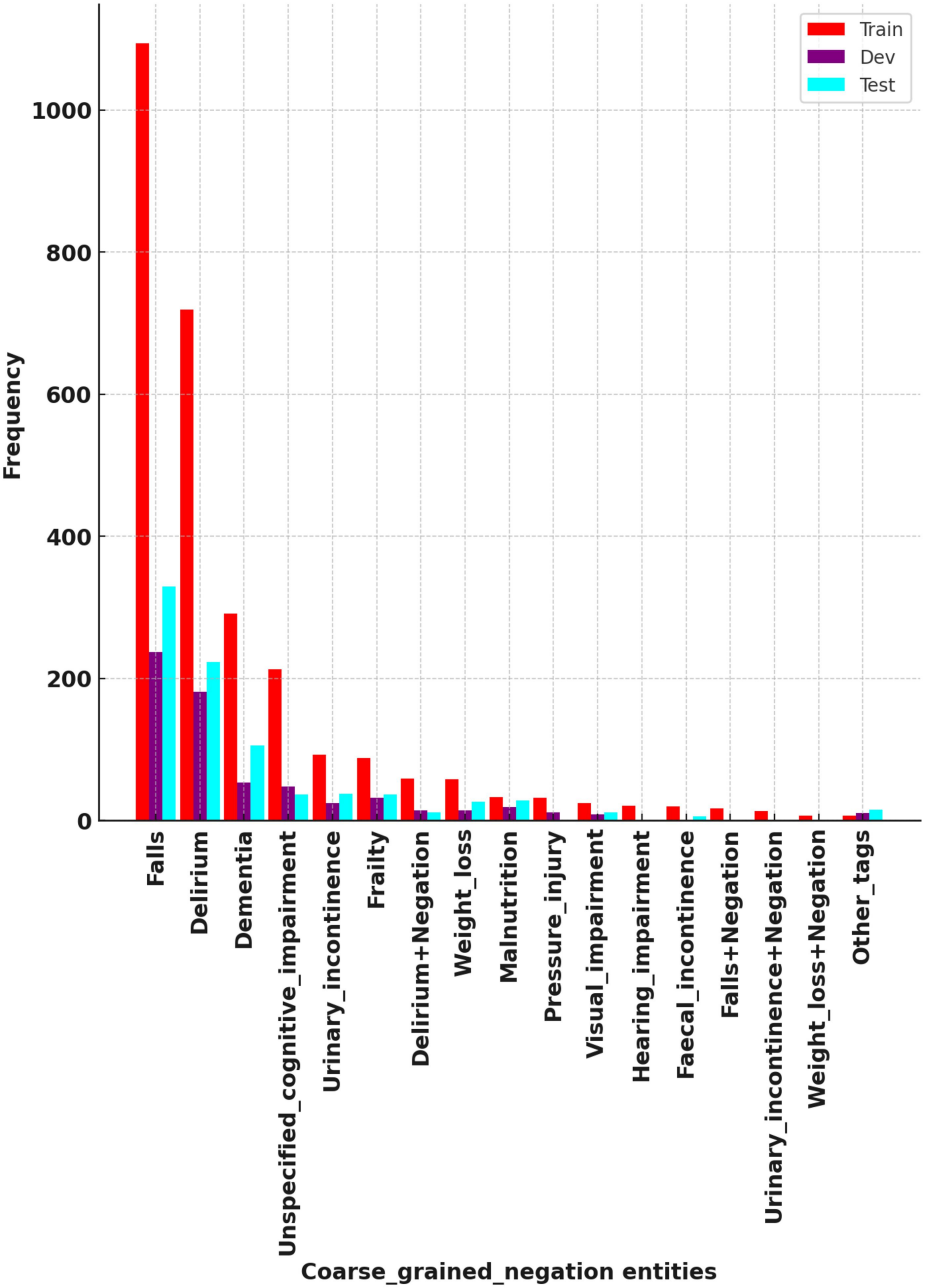
Distribution of coarse-grained GS entity labels with negation across the training, development and test sets. This setting retains the 12 core GS categories and distinguishes negated from non-negated mentions. The figure shows that negated GS mentions are less frequent than non-negated mentions, indicating that negation introduces additional label sparsity and may make the extraction task more challenging.

The coarse-grained annotation with negation captures the primary entities along with a single attribute—*negation*. In this case, the resulting labels would be *Falls* and *Delirium_negation*. Figures 4–6 illustrate the distribution of entity types for each granularity (fine-grained, coarse-grained and coarse-grained with negation) across the train, development and test datasets, respectively. Counts are mutually exclusive within each granularity level: each mention is counted once under its most specific label, and broader-granularity totals are obtained by collapsing these labels rather than by repeated counting.

### 4.3 Inter-annotator agreement

IAA was assessed on the doubly annotated pilot and gold-standard test subsets, in which both annotators independently labelled the same documents before disagreement resolution and adjudication. Table 3 presents the inter-annotator agreement (IAA) scores at both entity and document levels for the pilot and test datasets, illustrating considerable improvements in annotation consistency. The F1-score for entity-level agreement was computed using Bratiaa.^5^ In the pilot dataset, fine-grained annotations demonstrated a moderate agreement (*0.630*), whereas coarse-grained annotations shows a higher alignment (*0.762*). Incorporating annotation refinements led to notable improvements in the test dataset, with fine-grained agreement increasing to *0.721* and coarse-grained agreement reaching *0.823*. These enhancements underscore the benefit of using annotation guidelines on agreement levels but also the use of iteration as part of the annotation process which has previously be advocated by Alex et al. (42).

**Table 3:** IAA between the two annotators (at the entity and document level) using F1-score and Cohen’s kappa.

Corpus	Annotation type	Entity-level	Document-level	
		F1-score	F1-score	Cohen’s kappa
Pilot	Fine-grained	0.630	0.691	0.709
	Coarse-grained	0.762	0.853	0.851
	Coarse-grained + negation	0.762	0.860	0.850
Test	Fine-grained	0.721	0.767	0.789
	Coarse-grained	0.823	0.934	0.924
	Coarse-grained + negation	0.820	0.924	0.924

At the document level, the test dataset consistently exhibited superior performance compared to the pilot dataset across all annotation types. The fine-grained agreement improved from *0.691* to *0.767*, while coarse-grained agreement increased from *0.853* to *0.934*. Similarly, the agreement in the coarse-grained with negation approach rose from *0.860* to *0.924*. These findings confirm the effectiveness of pilot studies in refining annotation schemes and enhancing overall consistency. Notably, the higher IAA scores for document-level labelling reinforce its significance for clinical applications.

## 5 Evaluation

### 5.1 Methodology

The FlairNLP framework (43) was used for the modelling pipeline, including training, tagging and evaluation. In order to train a corpus using FlairNLP, the data must be formatted in CoNLL.^6^ The initial step therefore involved converting the annotated files from the BRAT .ann format into CoNLL, a flat token-level format. The original BRAT annotations allowed discontinuous and overlapping entities. However, because CoNLL-based sequence labelling requires a flat representation, these structures were simplified during conversion. Following the preprocessing strategy described by Stanovsky et al. (12) and Dai et al. (13), each discontinuous mention was replaced with the shortest contiguous span that fully covered all of its fragments, and overlapping mentions were merged into a single covering mention. Thus, in this paper, the approach from Dai et al. (13) was used for annotation flattening during BRAT-to-CoNLL conversion, not as a transition-based discontinuous NER model. Consequently, the NER experiments reported here should be interpreted as baseline sequence-labelling experiments over a flattened CoNLL-compatible representation of the original annotations. The original BRAT corpus preserves discontinuous and overlapping mention structures, but the models evaluated in this study do not directly model these structures.

Following this, we generated three versions of the dataset from each CoNLL file: fine-grained (retaining all transformed entities and attributes), coarse-grained (keeping only the primary entities) and coarse-grained with negation (including all entities along with the negation attribute). Each version was then used to train the same sequence labelling architecture with different input representation settings: static GloVe word embeddings (44), and four pretrained transformer language models providing contextualised token representations, namely: uncased and cased BERT (45),^7^ BioBERT (46) and BioClinicalBERT (47). In all cases, the input token representations were passed to the same downstream sequence tagger, allowing us to compare representation settings while keeping the tagging architecture fixed.

More specifically, all models were trained using a Long Short-Term Memory (LSTM)^8^ (48) plus Conditional Random Fields (CRFs)^9^ (49) sequence tagging architecture implemented in Flair. Thus, GloVe was used as a static embedding input to the LSTM-CRF tagger, whereas BERT, BioBERT and BioClinicalBERT provided contextualised token representations to the same LSTM-CRF tagging architecture. The training configuration included two Recurrent Neural Network (RNN)^10^ layers (50), a dropout rate of 0.16, a hidden size of 64 and a learning rate of 0.037. Due to computing constraints, i.e., limited access to a single shared GPU, we used a mini-batch size of 2, resulting in an average training time of approximately 36 h per model. Importantly, although this shared configuration enabled a controlled comparison across representation settings, we acknowledge that the reported learning rate is substantially higher than is typically used for end-to-end transformer fine-tuning and that the mini-batch size was small. These choices were partly driven by the compute constraints of working within a Trusted Research Environment with limited access to a shared GPU. The transformer-based results should therefore be interpreted as baseline results under a constrained training setup, rather than as fully optimised transformer performance.

We evaluated model performance on the test data using precision, recall and F1-score, calculated at both entity and document levels. At the entity level, a prediction was considered correct only if both the span boundaries and the entity label matched the gold standard annotation exactly (strict matching). For document-level evaluation, we assessed whether each geriatric syndrome was correctly labelled in the discharge summary, regardless of the number of mentions or their positions. This document-level evaluation is particularly relevant for clinical applications where the primary objective is to flag the presence of conditions for coding or screening purposes.

For fine-grained annotations, where entities include contextual attributes (e.g., Falls + Diagnosis + History_of), both the entity type and all attributes must match exactly for a prediction to be counted as correct. For coarse-grained annotations, only the primary entity type must match, while for coarse-grained + negation, both the entity type and the negation attribute (if present) must be correct. This hierarchical evaluation approach allows us to assess model performance at different levels of clinical detail and to quantify the trade-off between annotation granularity and extraction accuracy.

To compare the statistical significance of model performance, we calculated 95% confidence intervals (CIs) for entity-level precision and recall using the normal (Wald) approximation to the binomial distribution, implemented via the proportion_confint() function in statsmodels.stats.proportion.^11^ Because precision and recall are proportions of binary outcomes (correct vs. incorrect predictions), they can be modelled using a binomial framework. Although the normal approximation may be unreliable for small sample sizes or extreme proportions, our evaluation involved a sufficiently large number of entities. We additionally computed Wilson score intervals, which are known to provide more stable estimates in small-sample settings (51), and observed only negligible differences between the two methods. Therefore, we report the confidence intervals based on the normal approximation. We computed CIs only at the entity level, as document-level results are aggregated from entity-level predictions and do not represent independent model outputs.

### 5.2 Results

Table 4 presents a detailed comparison of the performance of five different models (GloVe, BERT-uncased, BERT-cased, BioBERT and Bio-Clinical BERT) in extracting geriatric syndromes (GS) from clinical discharge summaries. The results are reported at both entity and document level, across three annotation types: fine-grained, coarse-grained and coarse-grained + negation. The table highlights variations in precision, recall and F1-score, offering valuable insights into how different NLP models handle clinical text processing, with particular focus on annotation granularity and negation detection.

**Table 4:** Evaluation results: comparing the performance of 5 models on the extraction of entity-level GS annotations and labelling of document-level GS classifications, reporting precision, recall and F1-score for three annotation types.

Model	Annotation type	Entity-level			Document-level		
		Precision (95% CI)	Recall (95% CI)	F1	Precision	Recall	F1
Glove	Fine-grained	0.716 (0.697–0.734)	0.665 (0.646–0.684)	0.689	0.771	0.597	0.673
	Coarse-grained	0.877 (0.863–0.890)	0.837 (0.822–0.851)	0.856	0.910	0.843	0.875
	Coarse-grained + negation	0.872 (0.858–0.885)	0.834 (0.819–0.848)	0.853	0.899	0.829	0.862
BERT-uncased	Fine-grained	0.657 (0.638–0.677)	0.688 (0.669–0.706)	0.672	0.668	0.676	0.672
	Coarse-grained	0.861 (0.847–0.875)	0.888 (0.875–0.900)	0.875	0.843	0.893	0.867
	Coarse-grained + negation	0.858 (0.844–0.872)	0.878 (0.865–0.891)	0.868	0.825	0.880	0.852
BERT-cased	Fine-grained	0.678 (0.659–0.697)	0.708 (0.690–0.726)	0.692	0.690	0.703	0.696
	Coarse-grained	0.868 (0.854–0.881)	0.898 (0.886–0.910)	0.883	0.870	0.925	0.897
	Coarse-grained + negation	0.855 (0.841–0.869)	0.869 (0.856–0.882)	0.862	0.861	0.917	0.888
BioBERT	Fine-grained	0.645 (0.625–0.664)	0.672 (0.653–0.691)	0.658	0.668	0.637	0.652
	Coarse-grained	0.876 (0.862–0.889)	0.885 (0.872–0.898)	0.880	0.834	0.891	0.861
	Coarse-grained + negation	0.870 (0.856–0.883)	0.898 (0.886–0.910)	0.884	0.809	0.872	0.839
BioClinicalBERT	Fine-grained	0.665 (0.646–0.684)	0.702 (0.684–0.720)	0.683	0.686	0.681	0.684
	Coarse-grained	0.873 (0.860–0.886)	0.894 (0.882–0.906)	0.883	0.869	0.912	0.890
	Coarse-grained + negation	0.881 (0.868–0.894)	0.895 (0.883–0.907)	0.888	0.856	0.904	0.880

#### 5.2.1 Entity-level results

Entity-level extraction focuses on accurately identifying and categorizing GS within clinical discharge summaries while preserving annotation granularity. For fine-grained annotation, BERT-cased achieved the highest F1-score (0.692), while BioBERT performed the worst (0.658). Fine-grained annotation retains additional attributes such as history, suspected, referral and negation, making classification more challenging. The lower performance is expected due to the complexity of detailed entity recognition.

Based on the 95% CIs for precision and recall reported in Table 4, all five models show overlapping intervals for both metrics in the entity-level extraction setting. The overlap suggests that there is no strong evidence that any single model outperforms the others on these measures. Although BERT-cased achieved the highest fine-grained entity-level F1-score (0.692), GloVe performed very similarly (0.689) and outperformed the other transformer variants in this setting. This result is noteworthy, as transformer models are often expected to dominate clinical NLP tasks. However, the overlapping 95% confidence intervals for precision and recall suggest that these differences are modest and do not provide strong evidence that one model clearly outperforms the others in the fine-grained setting. A likely explanation is that fine-grained GS extraction is a low-resource and high-sparsity task, with many rare syndrome-attribute combinations that limit the benefit of contextual fine-tuning. In addition, the computational constraints of the trusted research environment limited hyperparameter optimisation which may have affected transformer performance. Under these conditions, simpler static embeddings such as GloVe, combined with a BiLSTM-CRF architecture, may remain competitive, especially when classification depends strongly on local lexical cues. This further indicates that the fine-grained task is inherently challenging, with performance differences between models remaining relatively modest.

The close performance of GloVe and BERT-cased in the fine-grained entity-level setting should be interpreted cautiously. It should not be taken as evidence that static embeddings are generally comparable to optimally tuned transformer models for GS extraction. Rather, this result may reflect the constrained training configuration used in this study, including the small mini-batch size and non-standard learning rate for transformer fine-tuning, together with the intrinsic difficulty of the fine-grained task. Fine-grained annotation requires exact span matching and exact matching of contextual attributes, which increases label sparsity and makes the task more sensitive to annotation complexity.

In the coarse-grained annotation, both BERT-cased and Bio-Clinical BERT achieved the highest F1-score (0.883). These results suggest that focusing only on the presence of GS, without additional attributes, allows models to perform with higher precision and recall. BioBERT followed closely behind with an F1-score of 0.880, reinforcing its strong performance in biomedical NLP.

The 95% CIs for precision and recall in the coarse-grained setting are relatively narrow, indicating low variance and a more stable evaluation compared with the fine-grained entity-level task. For precision, the CIs of all five models show substantially overlapping intervals, suggesting no clear statistical difference among them. In contrast, for recall, the BERT-based models show higher intervals compared with the GloVe model. This pattern indicates a modest but consistent recall advantage for transformer-based models in coarse-grained GS detection.

For coarse-grained + negation, Bio-Clinical BERT achieved the highest F1-score (0.888). Overall, adding negation resulted in performance that remained broadly comparable to the coarse-grained setting, rather than showing a uniform improvement across models. In particular, BioBERT and BioClinicalBERT showed small gains when negation was included, whereas GloVe, BERT-uncased and BERT-cased showed slight decreases. This pattern suggests that negation can be incorporated without a major reduction in performance overall, but its effect is model-dependent rather than consistently beneficial. The fact that all models maintained an F1-score above 0.85 indicates that negation is handled reasonably effectively, with domain-specific biomedical models appearing slightly more robust in this setting.

The 95% CIs for precision and recall in the coarse-grained + negation setting largely overlap across all five models. Similar to the entity-level analysis, this overlap indicates that there is no strong statistical evidence that any single model clearly outperforms the others.

#### 5.2.2 Document-level results

Document-level evaluation aggregates GS mentions across entire discharge summaries, reducing inconsistencies caused by position-dependent entity extraction. In fine-grained annotation, BERT-cased again achieved the highest F1-score (0.696). While document-level evaluation reduces the errors compared to entity-level processing, fine-grained annotation remains difficult due to the additional attributes involved.

For both coarse-grained annotation and coarse-grained + negation category, BERT-cased maintained the highest performance, reaching an F1-scores of 0.897 (for coarse-grained) and 0.888 (for coarse-grained + negation). This demonstrates the effectiveness of document-level aggregation with a considerable higher performance by mitigating entity recognition inconsistencies.

Document-level annotation conflicts were minimal (below 5 across the entire dataset) and therefore had negligible impact on model performance. We consequently retained these instances in the data.

#### 5.2.3 Results for individual entities

**Figure 7:**
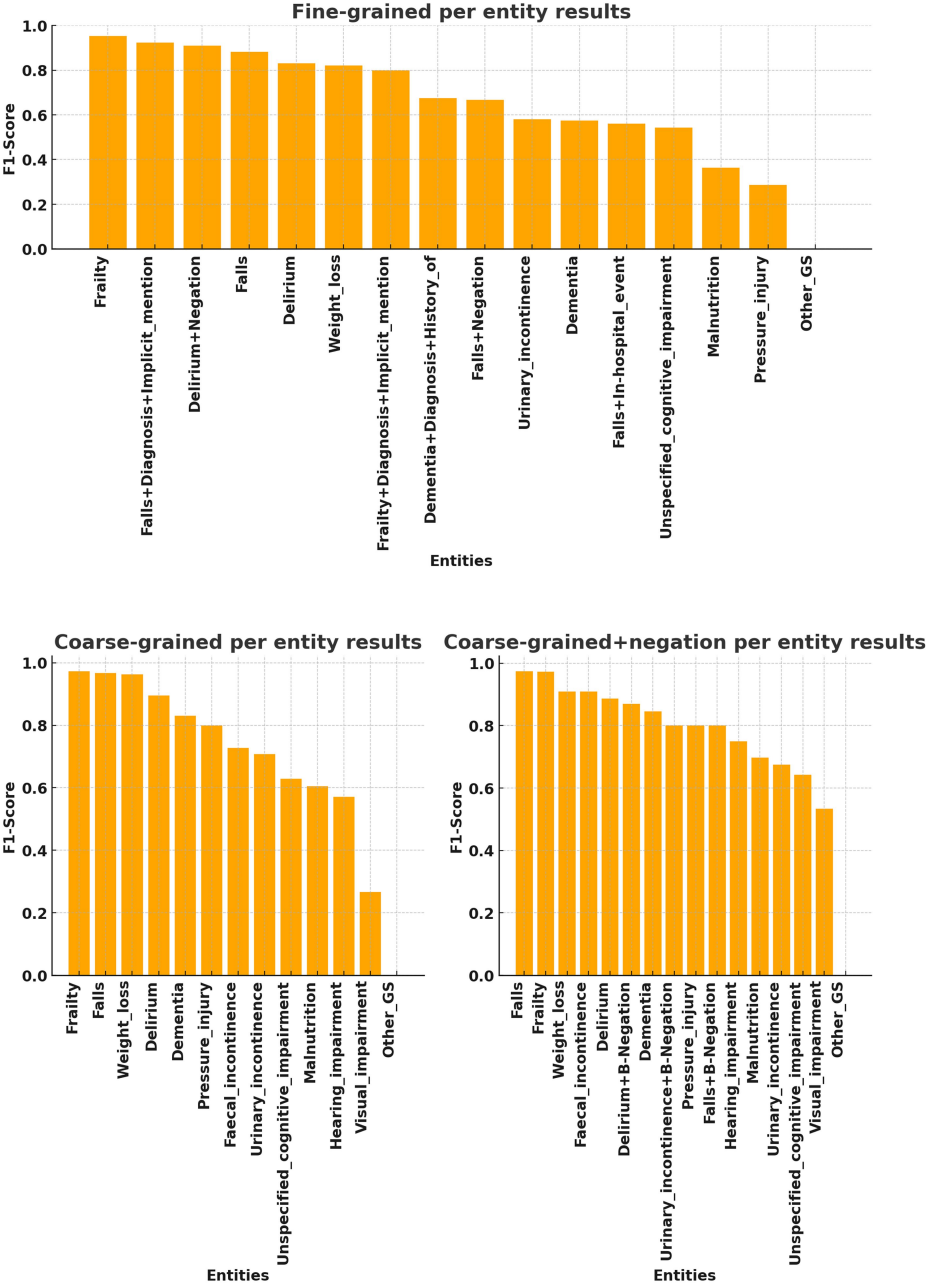
Results per entity type and granularity at the entity level.

For each granularity type, we use the best-performing model: BERT-cased in fine-grained analysis and BioClinicalBERT for both coarse-grained and coarse-grained + negation analysis. Figure 7 illustrates three bar charts presenting a comparative evaluation of F1-scores across different entities and granularities.

These per-entity results further show that dataset composition strongly influenced model outcomes. Syndromes with higher representation and more explicit lexical cues, such as frailty, falls and delirium, achieved the highest F1-scores across settings. In contrast, lower-performing categories such as malnutrition, pressure injury and visual impairment were more sparsely represented and often expressed with greater linguistic variability, making them harder for the model to learn reliably. This effect was even stronger in the fine-grained setting, where rare context combinations further fragmented the data.

For the fine-grained annotation, we obtained high F1-scores for entity types such as Frailty (0.952), Falls + Diagnosis + Implicit_mention (0.923) and Delirium + Negation (0.909). The recognition of these entities suggests that the model effectively identifies their key features within the dataset. Falls (0.882) and Delirium (0.831) also show relatively robust performance, reflecting their distinct and recognisable characteristics in the dataset. On the lower end, Malnutrition (0.364) and Pressure_injury (0.286) show weak performance. The lower F1 scores are attributed to a lack of sufficient training data. Finally, the Other_GS category (78 cases) represents instances where the model failed (F1-score = 0). This indicates challenges in detecting specific entity types and suggests that some categories remain under-represented or difficult to classify accurately. The Other_tags category includes entities like *Urinary_incontinence* + *Diagnosis* + *Implicit_mention* or *Weight_loss* + *Diagnosis* + *History_of* that appear in the test dataset just once.

For the coarse-grained annotation, Frailty (0.973), Falls (0.968) and Delirium (0.896) continue to show high performance, consistent with the fine-grained results. A noticeable improvement is seen in Malnutrition (0.605) compared to the fine-grained results. This suggests that merging similar entities helps the model achieve better results. Visual impairment (0.267) remains a low-performing category, indicating persistent difficulty in detecting this GS accurately. Finally, unlike the fine-grained dataset, there are no instances of Other_GS (0 cases) in the coarse-grained annotation. This suggests that broader categories ensure that all entities receive some degree of classification, reducing the number of completely unrecognised cases.

Finally, for the coarse-grained + negation annotations, the top-performing categories remain consistent, with Falls (0.974), Frailty (0.973) and Delirium (0.887). The model successfully differentiates these entities even when accounting for negation. Delirium + Negation (0.870) suggests that the model effectively recognises cases where the presence of delirium is negated in the text. This demonstrates that the model can interpret negation cues and adjust classification accordingly. Visual impairment results in higher performance for this model, reaching an F1-score of 0.533. The higher score indicates that incorporating negation handling improves entity recognition, by helping the model distinguish between confirmed and negated cases. The number of Other_GS category is just 3 (the model struggles to detect *Unspecified_cognitive_impairment* + *Negation* appearing twice and *Weight_loss* + *Negation* appearing once within the test dataset).

These per-category results should be interpreted cautiously because the dataset is highly imbalanced across geriatric syndrome labels. Frequent and lexically explicit categories, such as Falls, Frailty and Delirium, provide substantially more training evidence and therefore achieve stronger F1-scores. By contrast, low-frequency categories such as Pressure injury and Malnutrition have limited training support and greater linguistic variability. Since no class weighting, focal loss, oversampling or other imbalance-mitigation strategy was applied in the present baseline experiments, the low F1-scores for these categories should be understood primarily as reflecting data scarcity and label sparsity under the observed corpus distribution, rather than as a definitive measure of the models’ ability to recognise these syndromes.

## 6 Error analysis

To evaluate the performance of our models, we apply them to synthetic examples, which were created as part of our annotation guidelines development. Table 5 displays the different GS extracted (at both entity and document levels) for each level of granularity compared to gold annotations for one synthetic report presented in Figure 2. For each granularity type, we use the best-performing model defined in Section 5.2.3.

**Table 5:** Some examples. Values in parentheses indicate the model’s confidence score for the predicted label (a probability-like score in [0,1] returned by Flair for each extracted entity span).

Example	Annotation type	Entity-level	Document-level
Example 1	Fine-grained	[Span[7:8]: “frail”/Frailty (0.7771)	Frailty
		Span[15:16]: “confusion”/Delirium (0.8843)	Delirium
		Span[18:19]: “fall”/Falls (0.9998)	Falls
		Span[44:45]: “on”/Falls (0.9975)	Urinary_incontinence
		Span[66:69]: “incontinent of urine”/Urinary_incontinence (0.9879)	Falls + Diagnosis + Implicit_mention
		Span[74:75]: “confused”/Delirium (0.9946)	Weight_loss
		Span[76:77]: “disoriented”/Delirium (0.4458)	Malnutrition
		Span[85:86]: “falling”/Falls + Diagnosis + Implicit_mention (0.429)	Falls + In-hospital_event
		Span[129:131]: “weight loss”/Weight_loss (0.9359)	
		Span[149:150]: “dieticians”/Malnutrition (0.9297)	
		Span[154:155]: “fall”/Falls + In-hospital_event (0.9983)	
	Coarse-grained	Span[7:8]: “frail”/Frailty (0.9986)	Frailty
		Span[15:16]: “confusion”/Delirium (0.9998)	Delirium
		Span[18:19]: “fall”/Falls (1.0)	Falls
		Span[66:69]: “incontinent of urine”/Urinary_incontinence (0.9851)	Urinary_incontinence
		Span[74:75]: “confused”/Delirium (0.9992)	Weight_loss
		Span[76:77]: “disoriented”/Delirium (0.9997)	Malnutrition
		Span[85:86]: “falling”/Falls (1.0)	
		Span[120:121]: “delirium”/Delirium (0.9996)	
		Span[129:131]: “weight loss”/Weight_loss (0.9929)	
		Span[149:150]: “dieticians”/Malnutrition (0.9346)	
		Span[154:155]: “fall”/Falls (1.0)	
(Figure 2)	Coarse-grained + negation	Span[7:8]: “frail”/Frailty (0.9995)	Frailty
		Span[15:16]: “confusion”/Delirium (0.9991)	Delirium
		Span[18:19]: “fall”/Falls (1.0)	Falls
		Span[42:47]: “her lying on the floor”/Falls (0.959)	Urinary_incontinence
		Span[66:69]: “incontinent of urine”/Urinary_incontinence (0.9901)	Weight_loss
		Span[74:75]: “confused”/Delirium (0.9992)	Malnutrition
		Span[76:77]: “disoriented”/Delirium (0.9994)	
		Span[85:86]: “falling”/Falls (0.9987)	
		Span[120:121]: “delirium”/Delirium (0.9989)	
		Span[129:131]: “weight loss”/Weight_loss (0.9975)	
		Span[149:150]: “dieticians”/Malnutrition (0.9868), Span[154:155]	
		“fall”/Falls (1.0)	

Higher values correspond to higher confidence.

The tagging of this example aligns well with the overall results, as most coarse-grained entities were detected at the entity level and all were captured at the document level. The only exception is related to the discontinuous entity (“find” …“on the floor”) that was partially detected (“on”) as a fall on the entity level. However, the models exhibited difficulty in identifying the suspected attributes, as well as in detecting the negation of *delirium*. This can be primarily attributed to the limited number of instances of these categories in the training data. More broadly, these errors suggest that model performance was affected by the interaction between data sparsity and annotation complexity. Labels that were both rare and context-dependent, such as suspected or negated mentions, were harder to detect than common syndromes expressed in more direct language.

For instance, the dataset contains only nine instances of *Malnutrition_Diagnosis_Suspected* and 58 instances of *Delirium_Negation*, which limits the model’s ability to learn these patterns effectively. Moreover, as the focus of this study is to highlight the dataset and annotation guidelines, we did not explore techniques such as data balancing or adjusting class weights to mitigate the effects of underrepresented categories. Addressing these limitations remains an important objective for future work.

## 7 Limitations

First, the corpus exhibits a markedly imbalanced distribution of geriatric syndromes and associated attributes. Several categories—most notably pressure injury and visual impairment—are under-represented relative to more frequently documented syndromes such as falls and delirium. This imbalance likely affected model performance across categories: classes with higher support generally achieved more stable and higher scores, whereas rarer syndromes and fine-grained attribute combinations (e.g., suspected/referral/negated variants) produced noisier estimates and lower performance. Figures 4–6 report corpus-level counts per syndrome (and, per attribute for Figure 2 and using negation for Figure 3).

Second, the corpus remains relatively small—just over two thousand discharge summaries (2,040) from a specific cohort—drawn from discharge summaries from a single regional health system. As a result, documentation style, terminology, and syndrome prevalence may not reflect other institutions, EHR systems, countries, note types, or patient populations, and the reported performance may not fully generalise beyond this setting. We therefore view the current release as a foundational resource and emphasise the need for external validation and expansion. In particular, the annotated MIMIC discharge summaries (described in our future work) will serve both as an independent test set to quantify cross-domain generalisability—by training on one dataset and evaluating on the other in the secure environment—and, based on the obtained results and error analysis, as a potential source for enriching and expanding this dataset (e.g., improving coverage of rarer syndromes/attributes) while maintaining strict separation of training and evaluation data.

Third, the train, development and test splits were performed at the document level rather than at the patient level. Although the corpus contains discharge summaries originating from a cohort of 200 patients, the research team only had access to de-identified discharge summaries and did not have access to persistent patient identifiers or any document-to-patient linkage information. It was therefore not possible to ensure that all discharge summaries belonging to the same patient were assigned to the same split. This introduces a potential risk of data leakage, as documents from the same patient may have appeared in different splits, and may have led to optimistic performance estimates, particularly for document-level evaluation where repeated clinical histories or similar documentation patterns could be easier to recognise. Future work should address this limitation by performing patient-level splitting and patient-level external validation where governance arrangements permit access to linkage information.

Fourth, although the annotation scheme and original BRAT corpus explicitly capture discontinuous and overlapping entities, the baseline modelling experiments do not directly model these structures. For compatibility with FlairNLP and the CoNLL sequence-labelling format, discontinuous mentions were converted to shortest covering spans and overlapping mentions were merged during preprocessing, following the flattening strategy described by Stanovsky et al. (12) and Dai et al. (13). This flattening simplifies the task and may affect downstream NER evaluation, particularly for clinical expressions whose meaning depends on non-contiguous evidence or overlapping syndrome labels. The modelling results should therefore be interpreted as an initial exploitation of the dataset rather than a full evaluation of its discontinuous and overlapping annotation structure.

Fifth, the transformer-based models were not extensively hyperparameter-tuned. The learning rate and mini-batch size used in this study were constrained by the computational environment available within the Trusted Research Environment, including limited access to a shared GPU. We acknowledge that the learning rate is higher than is typically used for end-to-end transformer fine-tuning and that the small mini-batch size may have under-optimised the transformer-based models. Therefore, the comparison between GloVe and transformer-based embeddings should be interpreted as an initial baseline comparison rather than a definitive assessment of model capability. Because the project funding has ended and access to the Trusted Research Environment is no longer available, we were unable to rerun additional hyperparameter optimisation experiments for this revision. Also, because a single shared training configuration was used across representation settings, the transformer-based models may not have benefited from the lower learning rates and tuning strategies typically used in end-to-end BERT fine-tuning.

Sixth, another limitation concerns class imbalance. The distribution of geriatric syndrome mentions is highly skewed, with frequent categories such as Falls, Frailty and Delirium having substantially more training instances than rare categories such as Hearing impairment, Pressure injury and Malnutrition. The present baseline experiments did not use class weighting, focal loss, oversampling or targeted sampling to mitigate this imbalance. As a result, low F1-scores for rare categories should be interpreted primarily as reflecting limited training data, sparse contextual combinations and linguistic variability, rather than as definitive evidence that these syndromes cannot be modelled effectively. Future work should investigate imbalance-aware training strategies, including loss re-weighting, focal loss, oversampling of rare categories, threshold calibration and targeted annotation expansion for under-represented syndromes.

Seventh, this study was conducted within a Trusted Research Environment (TRE), which imposed certain constraints on model choices, data linkage and fairness evaluation. We focused on efficient model architectures, and it was not possible to deploy generative AI models such as GPT-4 or Mistral. Hyperparameter optimisation was also not feasible given the computational requirements. More importantly for fairness analysis, the clinical data controllers did not allow patient identifiers to be linked to documents as part of this project because of disclosure and identifiability concerns. We therefore could not examine heterogeneity in documentation practices or evaluate model performance across demographic subgroups.

This is an important limitation. Demographic categories of concern for future evaluation include age strata within the older-adult cohort, sex or gender, ethnicity, socioeconomic deprivation, preferred language or interpreter need, disability status, care-home or residential status and geography or service area. These categories are relevant because clinical documentation is not a neutral record of patient state: previous studies have shown that documentation patterns and the use of stigmatizing or negative language in EHR notes may vary by patient race and ethnicity, and that such language can transmit bias between clinicians or influence subsequent care (52–55). In the context of GS extraction, such differences could affect both annotation prevalence and model performance. For example, falls, frailty, cognitive impairment or continence problems may be documented differently depending on a patient’s age, ethnicity, socioeconomic context, living situation or communication needs.

When governance arrangements permit appropriate linkage to demographic metadata, future work should evaluate fairness at both corpus and model levels. At the corpus level, we will compare GS mention prevalence, note length, documentation density, contextual attribute use and annotation uncertainty across demographic groups. At the model level, we will report subgroup-specific precision, recall and F1-score for both entity-level extraction and document-level labeling, examine false-positive and false-negative rates by subgroup, and assess whether performance differences persist after accounting for syndrome prevalence and note length. Where sample sizes are small, we will use cautious aggregation, confidence intervals and disclosure-control procedures rather than reporting unstable subgroup estimates. These analyses will be conducted only within approved governance frameworks and with appropriate safeguards to avoid re-identification.

## 8 Future work

A key next step is to extend and validate our annotation scheme beyond the current NHS discharge summaries by applying the same methodology to the MIMIC-IV discharge summaries. This extension is not a simple transfer: our initial work on MIMIC-IV has been to expand the annotation guidelines to account for features that occur frequently in MIMIC-IV discharge letters, where some elements are recurring and appear to be structured or part of a template. These segments can contain clinically valuable information about the patients’ mobility status and cognitive state at discharge, yet may also introduce ambiguity or apparent contradictions with the narrative interpretation of the letter. We therefore propose specific guidance for annotating these recurring/template-like elements to ensure consistency and to reduce systematic label noise when scaling annotation.

A further priority is to exploit the discontinuous and overlapping entity annotations more directly. In the present paper, these annotations were flattened for compatibility with FlairNLP and CoNLL-based sequence labelling, but the original BRAT annotations preserve the full span structure. Future work will therefore evaluate modelling approaches designed for discontinuous and overlapping named entity recognition, including span-based, hypergraph-based or transition-based models. This is particularly relevant to our ongoing extension of the annotation scheme to MIMIC-IV discharge summaries, where we aim to examine these complex clinical mention structures in greater detail.

Because MIMIC-IV discharge summaries are typically longer and more heterogeneous, we will also scale the annotation workflow by involving a larger group of annotators and implementing a strengthened adjudication process (e.g., dual annotation on a larger calibration subset, regular disagreement meetings and periodic clinical review). Involving additional clinical experts will help refine edge cases (e.g., implicit mentions, conflicting template statements and borderline cognitive descriptors), and will support iterative updates to the guideline and examples.

A further priority is demographic fairness evaluation. Subject to governance approval, future work will link documents to protected and clinically relevant demographic metadata within the secure environment, including age strata, sex or gender, ethnicity, socioeconomic deprivation, preferred language or interpreter need, disability status, care-home or residential status and geography or service area. This will allow us to assess whether GS documentation patterns and model errors differ across patient groups. We will evaluate subgroup-specific precision, recall and F1-score, compare false-positive and false-negative rates, and examine whether observed differences are driven by documentation density, syndrome prevalence, note length or contextual attribute sparsity. These analyses will be designed with disclosure-control safeguards, especially for small or intersecting demographic groups.

In parallel, we will explore methods to improve performance on rare entities and fine-grained attribute combinations, which are currently impacted by data imbalance. Several strategies could mitigate imbalance in future iterations. At the modelling level, we will explore (i) loss re-weighting/class weights (e.g., inverse-frequency weighting or focal loss) to reduce bias toward majority classes; (ii) sampling-based approaches such as over-sampling rare syndromes or under-sampling dominant ones; and (iii) threshold calibration and macro-averaged model selection criteria to prevent optimisation being dominated by the most common categories. At the data level, we will investigate (iv) targeted annotation expansion by prioritising documents likely to contain under-represented syndromes (e.g., via keyword/ICD triage within governance constraints); and (v) data augmentation for low-frequency entities, such as synonym/abbreviation substitution, controlled span swapping, and back-translation-style paraphrasing, applied conservatively to preserve clinical meaning and evaluated with clinician review and strict held-out testing to avoid artificial performance gains.

As access to local trusted research environments can limit deployment of large generative models, MIMIC-IV also provides an opportunity to evaluate large language model (LLM) and hybrid extraction approaches under fewer operational constraints. We plan to investigate prompt-based and weakly supervised methods for automatic GS extraction, and to compare them with fine-tuned transformer baselines.

Finally, we aim to maximise downstream impact by leveraging the fact that MIMIC-IV includes rich structured data alongside the discharge summaries. Once GS annotations are available, we will study how geriatric syndromes interact with multimorbidity, clinical trajectories, outcomes and resource use. To facilitate broader research, and recognising that obtaining restricted datasets can require lengthy application processes, we plan to release the annotated MIMIC-IV-derived resource (guideline + extensions and derived labels where permitted) via PhysioNet as early as feasible, enabling reproducibility and accelerating research on geriatric syndromes in critical care and hospital populations.

## 9 Conclusion

This study presents a manually annotated dataset for extracting Geriatric Syndromes from clinical text, specifically focusing on discharge summaries of older patients. Unlike previous research, our dataset is designed for clinical applications, ensuring high annotation accuracy through expert collaboration. A key contribution of this work is the detection of discontinuous and overlapping entities, a challenge often overlooked in prior studies. We also evaluate multiple machine learning models to benchmark their performance, providing valuable insights for future clinical NLP research. The dataset is available upon request from Dataloch, ensuring controlled access for further study. Additionally, as future work, we plan to assess cross-domain generalisation by applying our annotation guidelines to the MIMIC-IV dataset (35). Inspired by the approach in (56), which introduced a multi-domain benchmark for AE detection, this evaluation will help determine how well models trained on Dataloch generalise to MIMIC-IV and vice versa, further advancing GS detection in clinical settings.

## Code availability statement

The code used for preprocessing, conversion of BRAT annotations to CoNLL format, model training and evaluation does not contain patient data and can be made available upon request to the corresponding author, subject to institutional approval and any required export checks from the secure research environment. Where possible, we will also make non-sensitive scripts and configuration files available in a public repository. Trained model weights are not openly released at this stage because the models were trained within a trusted research environment on restricted clinical text.

## Data Availability

The annotated dataset created as part of this study is derived from de-identified NHS Lothian discharge summaries and is held within the DataLoch secure research environment. Because the data are based on clinical records, neither the raw discharge summaries nor the corresponding patient-level annotation files can be openly released. Access to the dataset is available to approved researchers following a successful application to DataLoch and completion of the required governance, information-governance and ethical approval processes. Further information about the access process is available via DataLoch (https://dataloch.org).

## Footnotes

^1^The dataset may be accessed through DataLoch (dataloch.org) following a successful application and approvals.

^2^DataLoch is a Scottish regional Trusted Research Environment (TRE).

^3^https://usher.ed.ac.uk/sites/default/files/atoms/files/acrc_healthcare_data_research.pdf.

^4^https://brat.nlplab.org/.

^5^https://github.com/kldtz/bratiaa/.

^6^https://universaldependencies.org/format.html.

^7^We evaluated both BERT-uncased and BERT-cased, which differ in how they treat letter case during pre-training. Uncased models lowercase the input and discard capitalisation information, whereas cased models preserve case (e.g., “Fall” vs “fall”). Preserving case can help in clinical text where capitalisation carries signal (acronyms, abbreviations and named entities), while uncased models can be more robust to inconsistent casing. We therefore report results for both to quantify the impact of casing on GS extraction.

^8^In theory, classic RNNs can keep track of arbitrary long-term dependencies in the input sequences. The problem with classic RNNs is computational (or practical) in nature: when training a classic RNN using back-propagation, the long-term gradients which are back-propagated can “vanish”, meaning they can tend to zero due to very small numbers creeping into the computations, causing the model to effectively stop learning. RNNs using LSTM units partially solve the vanishing gradient problem, because LSTM units allow gradients to also flow with little to no attenuation. However, LSTM networks can still suffer from the exploding gradient problem.

^9^CRFs are a class of statistical modeling methods often applied in pattern recognition and machine learning and used for structured prediction. Whereas a classifier predicts a label for a single sample without considering “neighbouring” samples, a CRF can take context into account.

^10^Recurrent neural networks (RNNs) are a class of artificial neural networks designed for processing sequential data, such as text, speech, and time series, where the order of elements is important. Unlike feedforward neural networks, which process inputs independently, RNNs utilise recurrent connections, where the output of a neuron at one time step is fed back as input to the network at the next time step. This enables RNNs to capture temporal dependencies and patterns within sequences.

^11^https://www.statsmodels.org/dev/generated/statsmodels.stats.proportion.proportion_confint.html.
