# Supplementary material for: Geriatric syndromes extraction from discharge summaries: a new dataset, annotation scheme and initial findings
Source: Front Digit Health. 2026 Jul 13;8:1591050. doi: 10.3389/fdgth.2026.1591050 (PMC13402457; doi:10.3389/fdgth.2026.1591050)
Supplement: Supplementary file 1 [file Supplementaryfile1.pdf]

# Geriatric Syndromes Extraction from Discharge Summaries: A New Dataset, Annotation Scheme and Initial Findings ( Supplementary Material / Appendix)

**Imane Guellil** [1,4], **Salomé Andres** [1], **Atul Anand** [1], **Bruce Guthrie** [1], **Fahrurrozi Rahman** [1], **Abul Hasan** [2,3], **Huayu Zhang** [1], **Honghan Wu** [5], **Beatrice Alex** [1,5]

[1] *Advanced Care Research Centre - Usher Institute. University of Edinburgh, Edinburgh, United Kingdom*
[2] *Institute of Health Informatics.University College London (UCL), London, United Kingdom,*
[3] *Nuffield Department of Primary Care Health Sciences, Oxford University, Oxford, United Kingdom,*
[4] *Department of Cancer and Genomic Sciences. University of Birmingham,*
[5] *School of Health & Wellbeing. University of Glasgow,*
[6] *School of Mathematical & Computer Sciences, Heriot-Watt University, Edinburgh, United Kingdom*

Correspondence*:
Imane Guellil
imane.guellil@ed.ac.uk

## APPENDIX

### Table comparing previous related works

Table 1 presents a summary of all related work relevant to this paper.

### Comparison of entity- and document-level results

When comparing entity-level and document-level results, a clear trend emerges: document-level annotation yields higher F1-scores across all models and annotation types. This pattern is particularly noticeable in coarse-grained and coarse-grained + negation categories, where document-level F1-scores exceed their entity-level counterparts. Fine-grained annotation increases specificity by encoding contextual attributes, but it also increases task complexity. The label space becomes larger and more imbalanced, and some attribute boundaries (e.g., history vs current; implicit mention vs risk/hypothetical) can be subtle in narrative clinical text. As a result, fine-grained evaluation is less forgiving: partial correctness at the entity level is penalised when attributes are mismatched. This finding indicates a need for further refinements, such as knowledge integration or fine-tuning on domain-specific data, to improve fine-grained GS classification.

**Table 1.** Summary of related work.

| Paper | Category | Approach | Datasets / lexicons | Sources | Annotation guidelines | Number annotators | Entities | Discontinuous entities | Overlapping entities | IAA reported | Model embedding / ontology | ML algorithm |
|---|---|---|---|---|---|---|---|---|---|---|---|---|
| Scharp et al. (2025) | Extraction | Multiclass classification (note level) | Visit notes & coordination notes | Home Healthcare agency | - | 2 | 8 urinary incontinence symptoms | - | - | - | Word2Vec, Phrase2Vec | NimbleMiner |
| Cheligeer et al. (2024) | Classification | Binary classification (note level) | Progress notes | 3 hospitals in Alberta | - | 2 | fall/no_fall | - | - | Cibger, Fleiss, Light Kappa | BERT, BioClinicalBERT, Bag of Words, TF-IDF | SVM, LR, DT, NN, Rule-based |
| Dormosh et al. (2023) | Classification | Binary classification (note level) | Primary care notes | Academic General Practitioner's Network at the Academic Medical Center | - | - | fall/no_fall | - | - | - | Top2Vec | LR with LASSO |
| Mishra et al. (2023) | Classification | Binary classification (note level) | Nurse notes, visit notes, progress notes | TigerPlace senior living facility | - | - | fall/no_fall | - | - | - | BioWordVec, GloVe | LSTM, GRU |
| Maclagan et al. (2023) | Classification + Extraction | Binary classification (patient level) | Consult notes, progress notes | Electronic Medical Records Primary Care | - | 1 for categories, 3 for review | dementia / no_dementia, 8 categories | - | - | - | Bag of Words, TF-IDF on 8 feature lists | LR with LASSO & Ridge, Gradient Boosting, NN |
| Shao et al. (2023) | Classification | Binary classification (patient level) | Clinical notes | Veterans Health Administration Corporate Data Warehouse | - | 3 for evaluation data | dementia / no_dementia | - | - | Cohen's Kappa | Latent Dirichlet Allocation (LDA) topics | SVM |
| Alkhalaf et al. (2023) | Classification + Extraction | Rule-based | Progress notes | 40 Residential Aged Care Facilities | - | 2 for extracted terms, 3 for extracted notes | 15 factors, malnutrition / no_malnutrition | - | - | - | - | Regular expression |
| Penfold et al. (2022) | Classification + Extraction | Binary classification | Clinical notes | Kaiser Permanente Washington Health Research Institute | - | 3 for note review | MCI/no_MCI, 42 concepts | - | - | - | Unique concept presence + symptom score, behaviour score, forgetful score | LR with LASSO |
| Fu et al. (2022) | Classification | Binary classification (sentence, note & patient level) | Clinical notes | Mayo Clinic Biobank | Yes | 2 | fall/no_fall | - | - | F1-Score | BERT, GloVe | Rule-based, CNN+BiLSTM, BERT, BERT+Rule-based |
| Wang et al. (2022) | Classification | Binary classification | Hospital admissions including lab test, diagnosis, clinical intervention, room transfer, clinical risk score, etc. | General Medicine Inpatient Initiative | - | 2 | delirium / no_delirium | - | - | - | TF-IDF, word count, n-gram | LR, NB, SVM, DT, RF, GB, XGBoost, MLP |
| Fu et al. (2020) | Classification | Binary & multiclass classification | Clinical notes | Mayo Clinic Biobank | - | 2 | delirium / no_delirium, definite, possible | - | - | F1-Score | - | Rule-based |
| Ge et al. (2022) | Classification | Binary classification (sentence level) | Clinical notes | 9 hospitals | - | 7 | delirium / no_delirium | - | - | Pairwise | Bag of unigrams & bigrams, BERT | SVM, LSTM, Transformer |
| Hane et al. (2020) | Classification + Extraction | Binary classification | Clinical notes | OptumLabs Data Warehouse | - | - | dementia / no_dementia, cognitive terms | - | - | - | Term clusters | LightGBM |
| Nakatani et al. (2020) | Classification | Binary classification | Nurse notes | NTT Medical Center Tokyo | - | - | fall/no_fall | - | - | - | Document Word Embedding Matrix | Markov Chain Monte Carlo |
| Lorenzoni et al. (2021) | Extraction | Clustering | Clinical notes | Local health authority in Italy | - | - | 3 fall patterns | - | - | - | Latent Dirichlet Allocation (LDA) topics | Hierarchical Clustering |
| Chen et al. (2019) | Extraction | Multiclass classification (patient & note level) | Clinical notes | Regional Medicare Advantage Health Maintenance Organization | - | 3 | 10 geriatric syndrome constructs | - | - | - | Linguistic features + ICD annotation | CRF |
| dos Santos et al. (2019) | Classification | Binary classification | Progress notes, incidence reports | A hospital in Porto Alegre, Brazil | - | 3 | fall/no_fall | - | - | - | Word2Vec, FastText, TF-IDF | LSTM, RF, SVM |
| Martin et al. (2021) | Classification | Multiclass classification | Clinical notes | University of Pennsylvania Health System | Yes | 2 annotators and 1 reviewer | 4 categories of frailty | - | - | - | Word2Vec, BioClinicalBERT, RoBERTa | Elastic Net Regression, RF, NN |
| Soysal et al. (2022) | Profiling | - | Clinical notes | South London and Maudsley NHS Foundation Trust (SLaM) | - | - | Weight loss & mortality, weight loss & emergency hospitalisation | - | - | - | - | General Architecture for Text Engineering |
| Du et al. (2024) | Classification | Binary classification | Clinical notes | Mass General Brigham's Enterprise Data Warehouse | - | 3 | yes, no, keywords for cognitive decline | - | - | Fleiss Kappa | GPT-4, Llama 2 | XGBoost, LSTM+FFNN, Prompt Engineering, Ensemble |

The performance differences across settings are also closely related to the characteristics of the dataset. In particular, fine-grained annotation creates a larger and sparser label space, because each syndrome may be combined with contextual attributes such as history, suspected, referral, negation and in-hospital onset. This increases class imbalance and makes learning more difficult, especially for rare label combinations. In addition, entity-level extraction is more sensitive to annotation complexity, including discontinuous and overlapping mentions, because the model must recover both the correct syndrome and its exact span. By contrast, document-level aggregation is less affected by local span errors, which helps explain why document-level results were consistently higher.

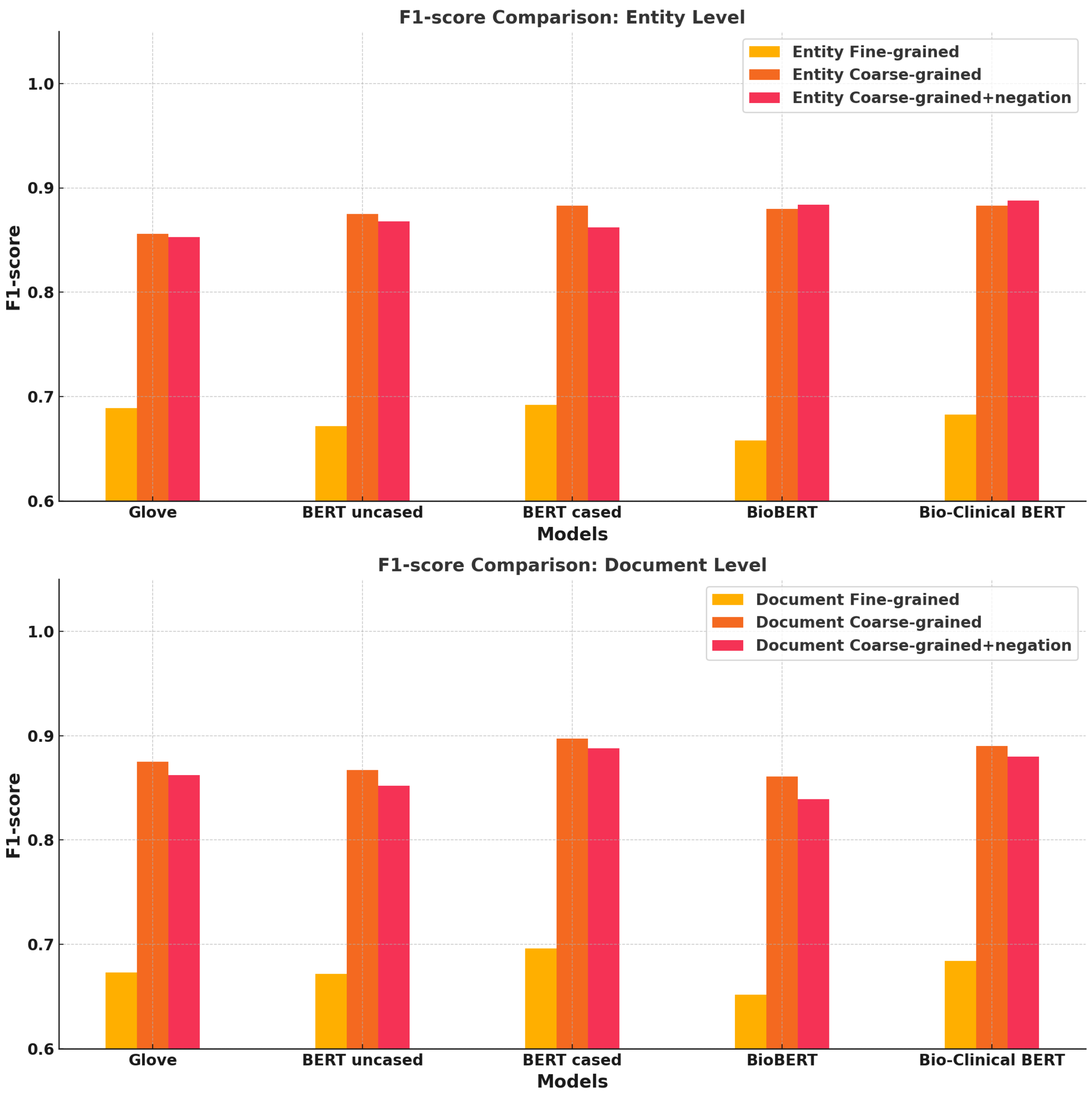


**Figure 1.** Comparison of the F1-score among the different models on the entity and the document level

In coarse-grained annotation, document-level aggregation yields higher precision and recall because it is less sensitive to missed mentions: a document is counted as a true positive if the syndrome is detected anywhere in the text, whereas entity-level evaluation requires identifying each mention. For example, the best-performing model, BERT-cased, achieved an F1-score of 0.883 at the entity-level and 0.897 at the document-level. Similarly, in coarse-grained + negation, document-level annotation consistently aligns with higher F1-scores across models.

Figure 1 compares the F1-scores cross different models at both levels, highlighting the overall trend that document-level annotation yields higher performance, particularly in coarse-grained and coarse-grained + negation categories, where models achieve consistently higher F1-scores compared to entity-level annotation. This figure also reinforces the strong performance of BERT-cased and Bio-Clinical BERT compared to other models, especially in structured annotation tasks, confirming their effectiveness in geriatric syndrome extraction from clinical texts.

## Additional synthetic examples

This section presents further synthetic examples

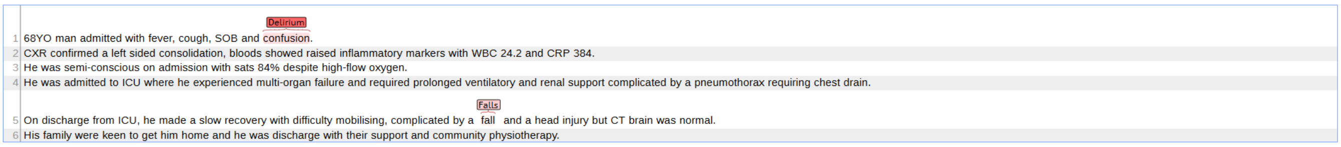


**Figure 2.** Example 1: A synthetic example annotated in BRAT includes the different entities and attributes.

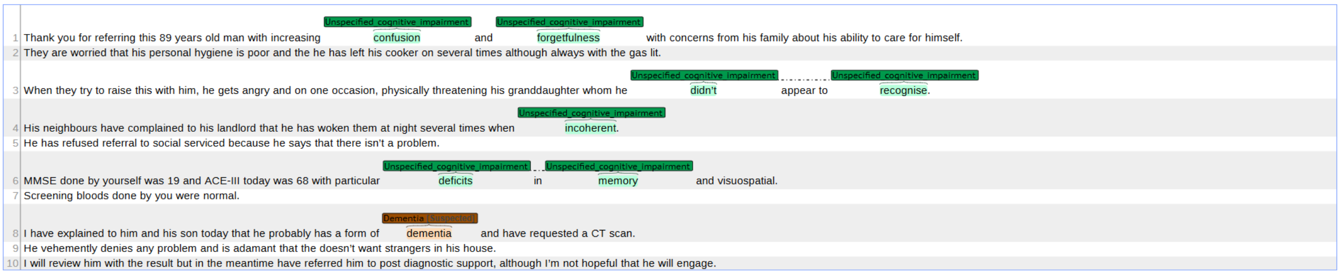


**Figure 3.** Example 2: A synthetic example annotated in Brat includes the different entities and attributes.

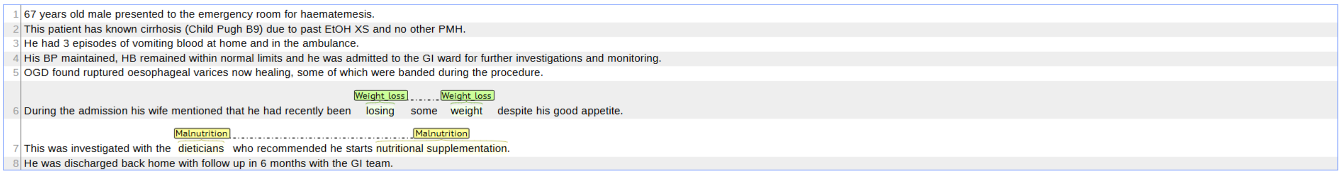


**Figure 4.** Example 3: A synthetic example annotated in BRAT includes the different entities and attributes.

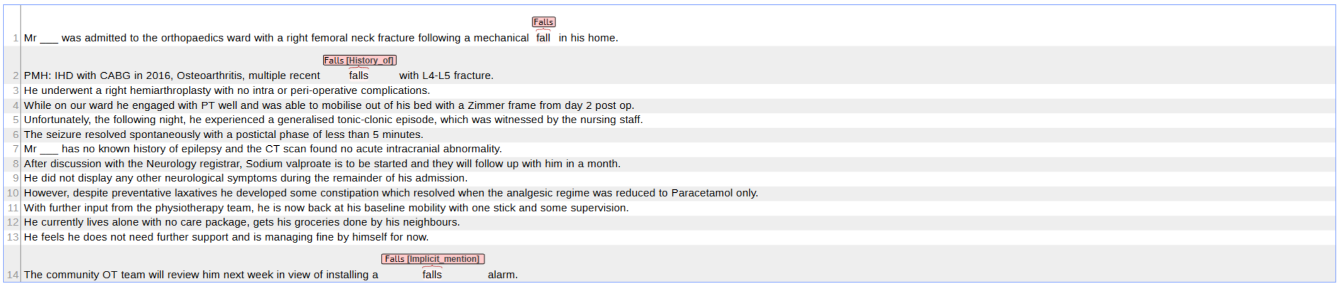


**Figure 5.** Example 4: A synthetic example annotated in BRAT includes the different entities and attributes.

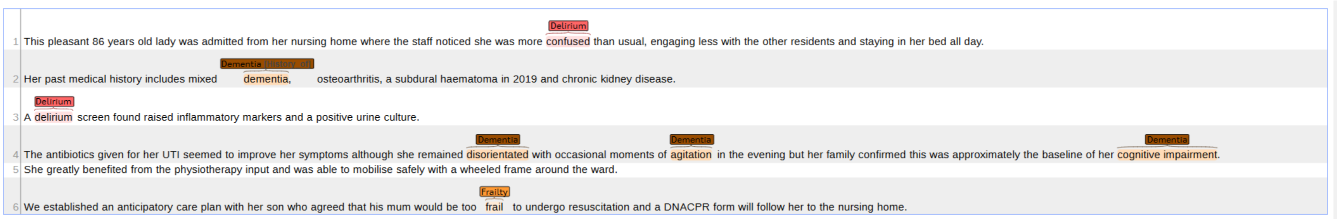


**Figure 6.** Example 5: A synthetic example annotated in BRAT includes the different entities and attributes.

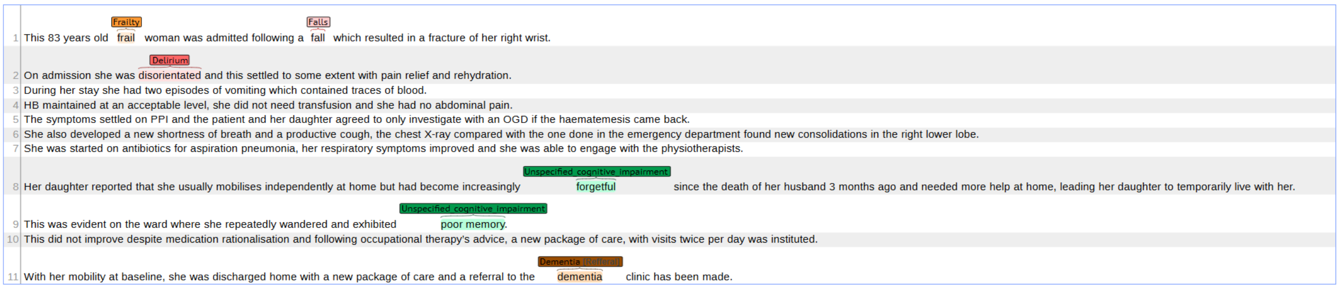


**Figure 7.** Example 6: A synthetic example annotated in BRAT includes the different entities and attributes.

## Coarse-grained pilot entity-level inter-annotator agreement (IAA) by label

The coarse-grained pilot entity-level agreement results suggest that annotation consistency was generally stronger for the more frequent and clinically explicit labels. In particular, Falls showed the highest support across both annotators (35 and 33 annotations, respectively) and also achieved the strongest non-trivial agreement (F1 = 0.912), followed by Dementia (16 and 12 annotations; F1 = 0.786) and Delirium (20 and 16 annotations; F1 = 0.667). Unspecified cognitive impairment, despite having moderate support (7 and 8 annotations), showed substantially lower agreement (F1 = 0.400), indicating that this category was more difficult to annotate consistently, likely because of broader semantic scope and less explicit textual cues. Several low-frequency categories, such as Faecal incontinence, Hearing impairment and Pressure injury, achieved perfect agreement, but these results should be interpreted cautiously because counts below 5 were suppressed for disclosure control and small sample sizes can inflate agreement estimates. Conversely, rare categories such as Frailty and Malnutrition showed zero agreement in the pilot phase, suggesting that low-frequency labels were more vulnerable to interpretive differences before guideline refinement. Overall, these results support the view that annotation consistency was more stable for frequent and lexically clearer entities, while rarer or conceptually broader categories showed greater variability.

## Coarse-grained test entity-level inter-annotator agreement (IAA) by label

The coarse-grained test entity-level agreement results suggest that annotation consistency was generally strongest for the more frequent and clinically explicit syndrome categories. Falls was the most common label in the doubly annotated test subset (327 and 311 annotations for the two annotators, respectively) and showed high agreement (F1 = 0.906). Similarly, Delirium (236 and 201 annotations) and Dementia (101 and 94 annotations) also achieved strong agreement, with F1-scores of 0.815 and 0.862, respectively. Frailty showed the highest agreement overall (F1 = 0.971), although with lower support than Falls, Delirium and Dementia. Weight loss also achieved strong agreement (27 and 25 annotations; F1 = 0.885). In contrast, categories such as Unspecified cognitive impairment (41 and 56 annotations; F1 = 0.474), Malnutrition (29 and 18 annotations; F1 = 0.553), Visual impairment (11 and 7 annotations; F1 = 0.556) and Pressure

**Table 2.** Coarse-grained pilot entity-level inter-annotator agreement (IAA) by label, with the corresponding number of annotations from Annotator 1 and Annotator 2. Values reported as $< 5$ are suppressed for statistical disclosure control.

| Label | Annotator 1 | Annotator 2 | IAA F1 |
|---|---|---|---|
| Delirium | 20 | 16 | 0.667 |
| Dementia | 16 | 12 | 0.786 |
| Faecal_incontinence | < 5 | < 5 | 1.000 |
| Falls | 35 | 33 | 0.912 |
| Frailty | < 5 | – | 0.000 |
| Hearing_impairment | < 5 | < 5 | 1.000 |
| Malnutrition | < 5 | – | 0.000 |
| Pressure_injury | < 5 | < 5 | 1.000 |
| Unspecified_cognitive_impairment | 7 | 8 | 0.400 |
| Urinary_incontinence | < 5 | < 5 | 0.750 |
| Visual_impairment | < 5 | < 5 | 0.667 |
| Weight_loss | < 5 | < 5 | 0.800 |

**Table 3.** Coarse-grained test entity-level inter-annotator agreement (IAA) by label, with the corresponding number of annotations from Annotator 1 and Annotator 2. Values reported as $< 5$ are suppressed for statistical disclosure control. Labels corresponding to attributes rather than coarse-grained GS categories are shown where present in the agreement output, but are not interpreted as core GS labels.

| Label | Annotator 1 | Annotator 2 | IAA F1 |
|---|---|---|---|
| Delirium | 236 | 201 | 0.815 |
| Dementia | 101 | 94 | 0.862 |
| Diagnosis | – | 156 | – |
| Faecal_incontinence | 8 | 6 | 0.714 |
| Falls | 327 | 311 | 0.906 |
| Frailty | 36 | 34 | 0.971 |
| GS | – | < 5 | 0.000 |
| Hearing_impairment | < 5 | < 5 | 0.750 |
| In-hospital_event | – | 20 | – |
| Low_confidence | – | < 5 | – |
| Malnutrition | 29 | 18 | 0.553 |
| Negation | – | 23 | – |
| Pressure_injury | < 5 | < 5 | 0.500 |
| Unspecified_cognitive_impairment | 41 | 56 | 0.474 |
| Urinary_incontinence | 41 | 35 | 0.632 |
| Visual_impairment | 11 | 7 | 0.556 |
| Weight_loss | 27 | 25 | 0.885 |

injury ($< 5$ and $< 5$ annotations; F1 = 0.500) showed lower agreement, suggesting that these entities were more difficult to annotate consistently, likely because of broader semantic scope, subtler contextual cues or greater lexical variation. Overall, the test-set analysis supports the view that annotation consistency was more stable for frequent and lexically clearer syndrome categories, while rarer or conceptually broader labels remained more challenging.

## CONFLICT OF INTEREST STATEMENT

The authors declare that the research was conducted in the absence of any commercial or financial relationships that could be construed as a potential conflict of interest.

## AUTHOR CONTRIBUTIONS

IG, BG and BA contributed to the conception and the design of the study. IG drafted the first version of the annotation guidelines. SA improved and enrich the annotation guidelines based on her clinical expertise. FR, IG, AH and HZ were involved in the collection and analysis of research papers in the related work section and FR synthesised these studies drafted the related work section. SA and BG proposed the first synthetic examples. IG set up the annotation tool and prepared the dataset for the annotation. SA annotated the whole dataset. SA is the first annotator of the gold dataset. AA is the second annotator of the gold dataset. IG developed, implemented and run the different experiments. FR computed the confidence interval scores and data statistics. IG drafted the manuscript. All the authors contributed to the article and approved the submitted version.

## FUNDING

This research was funded by the Legal & General Group (research grant to establish the independent Advanced Care Research Centre at University of Edinburgh). The funder had no role in conduct of the study, interpretation or the decision to submit for publication. The views expressed are those of the authors and not necessarily those of Legal & General.

This study/project (AIM-CISC) is funded by the National Institute for Health Research (NIHR). Artificial Intelligence and Multimorbidity: Clustering in Individuals, Space and Clinical Context (AIM-CISC) grant NIHR202639. The views expressed are those of the author(s) and not necessarily those of the NIHR or the Department of Health and Social Care.

## ACKNOWLEDGMENTS

This research has been assisted by the DataLoch service (reference: DL_2021_009) and received favourable ethical opinion under DataLoch's delegated Research Ethics authority (Reference: 17/NS/0027). DataLoch enables access to de-identified extracts of health care data from the South-East Scotland region to approved applicants: dataloch.org.

For MIMIC IV, the collection of patient information and creation of the research resource was reviewed by the Institutional Review Board at the Beth Israel Deaconess Medical Center, who granted a waiver of informed consent and approved the data sharing initiative.

The authors would like to warmly thank Richard Tobin (School of Informatics) for his amazing help in setting up BRAT. They would also like to thank the ACRC management team for their valuable support.

## DATA AVAILABILITY STATEMENT

The annotated dataset created as part of this study is derived from de-identified NHS Lothian discharge summaries and is held within the DataLoch secure research environment. Because the data are based on clinical records, neither the raw discharge summaries nor the corresponding patient-level annotation files can be openly released. Access to the dataset is available to approved researchers following a successful application to DataLoch and completion of the required governance, information-governance and ethical approval processes. Further information about the access process is available via DataLoch (`https://dataloch.org`).

## CODE AVAILABILITY STATEMENT

The code used for preprocessing, conversion of BRAT annotations to CoNLL format, model training and evaluation does not contain patient data and can be made available upon request to the corresponding author, subject to institutional approval and any required export checks from the secure research environment. Where possible, we will also make non-sensitive scripts and configuration files available in a public repository. Trained model weights are not openly released at this stage because the models were trained within a trusted research environment on restricted clinical text.

## REFERENCES

Alkhalaf, M., Zhang, Z., Chang, H.-C. R., Wei, W., Yin, M., Deng, C., et al. (2023). Malnutrition and its contributing factors for older people living in residential aged care facilities: Insights from natural language processing of aged care records. *Technology and Health Care* 31, 2267–2278. doi:10.3233/THC-230229

Cheligeer, C., Wu, G., Lee, S., Pan, J., Southern, D. A., Martin, E. A., et al. (2024). Bert-based neural network for inpatient fall detection from electronic medical records: Retrospective cohort study. *JMIR Med Inform* 12, e48995. doi:10.2196/48995

Chen, T., Dredze, M., Weiner, J. P., Hernandez, L., Kimura, J., and Kharrazi, H. (2019). Extraction of geriatric syndromes from electronic health record clinical notes: Assessment of statistical natural language processing methods. *JMIR Med Inform* 7, e13039. doi:10.2196/13039

Dormosh, N., Schut, M. C., Heymans, M. W., Maarsingh, O., Bouman, J., van der Velde, N., et al. (2023). Predicting future falls in older people using natural language processing of general practitioners' clinical notes. *Age and Ageing* 52, afad046. doi:10.1093/ageing/afad046

dos Santos, H. D., Silva, A. P., Maciel, M. C. O., Burin, H. M. V., Urbanetto, J. S., and Vieira, R. (2019). Fall detection in EHR using word embeddings and deep learning. In *2019 IEEE 19th International Conference on Bioinformatics and Bioengineering (BIBE)*. 265–268. doi:10.1109/BIBE.2019.00054

Du, X., Novoa-Laurentiev, J., Plasek, J. M., Chuang, Y.-W., Wang, L., Marshall, G. A., et al. (2024). Enhancing early detection of cognitive decline in the elderly: a comparative study utilizing large language models in clinical notes. *eBioMedicine* 109, 105401. doi:https://doi.org/10.1016/j.ebiom.2024.105401

Fu, S., Lopes, G. S., Pagali, S. R., Thorsteinsdottir, B., LeBrasseur, N. K., Wen, A., et al. (2020). Ascertainment of delirium status using natural language processing from electronic health records. *The Journals of Gerontology: Series A* 77, 524–530. doi:10.1093/gerona/glaa275

Fu, S., Thorsteinsdottir, B., Zhang, X., Lopes, G. S., Pagali, S. R., LeBrasseur, N. K., et al. (2022). A hybrid model to identify fall occurrence from electronic health records. *International Journal of Medical Informatics* 162, 104736. doi:https://doi.org/10.1016/j.ijmedinf.2022.104736

Ge, W., Alabsi, H., Jain, A., Ye, E., Sun, H., Fernandes, M., et al. (2022). Identifying patients with delirium based on unstructured clinical notes: Observational study. *JMIR Form Res* 6, e33834. doi:10.2196/33834

Hane, C. A., Nori, V. S., Crown, W. H., Sanghavi, D. M., and Bleicher, P. (2020). Predicting onset of dementia using clinical notes and machine learning: Case-control study. *JMIR Med Inform* 8, e17819. doi:10.2196/17819

Lorenzoni, G., Rampazzo, R., Buratin, A., Berchialla, P., and Gregori, D. (2021). Does the integration of pre-coded information with narratives improve in-hospital falls' surveillance? *Applied Sciences* 11. doi:10.3390/app11104406

Maclagan, L. C., Abdalla, M., Harris, D. A., Stukel, T. A., Chen, B., Candido, E., et al. (2023). Can patients with dementia be identified in primary care electronic medical records using natural language processing? *Journal of Healthcare Informatics Research* 7, 42–58. doi:10.1007/s41666-023-00125-6

Martin, J. A., Crane-Droesch, A., Lapite, F. C., Puhl, J. C., Kmiec, T. E., Silvestri, J. A., et al. (2021). Development and validation of a prediction model for actionable aspects of frailty in the text of clinicians' encounter notes. *Journal of the American Medical Informatics Association* 29, 109–119. doi:10.1093/jamia/ocab248

Mishra, A. K., Chappell, M. J., Emerson, S., and Skubic, M. (2023). Fall risk prediction in older adults using free-text nursing notes and medications in electronic health records. In *2023 45th Annual International Conference of the IEEE Engineering in Medicine & Biology Society (EMBC)*. 1–4. doi:10.1109/EMBC40787.2023.10341127

Nakatani, H., Nakao, M., Uchiyama, H., Toyoshiba, H., and Ochiai, C. (2020). Predicting inpatient falls using natural language processing of nursing records obtained from japanese electronic medical records: Case-control study. *JMIR Med Inform* 8, e16970. doi:10.2196/16970

Penfold, R. B., Carrell, D. S., Cronkite, D. J., Pabiniak, C., Dodd, T., Glass, A. M., et al. (2022). Development of a machine learning model to predict mild cognitive impairment using natural language processing in the absence of screening. *BMC Medical Informatics and Decision Making* 22, 129. doi:10.1186/s12911-022-01864-z

Scharp, D., Song, J., Hobensack, M., Palmer, M. H., Barcelona, V., and Topaz, M. (2025). Applying natural language processing to understand symptoms among older adult home healthcare patients with urinary incontinence. *Journal of Nursing Scholarship* 57, 152–164. doi:https://doi.org/10.1111/jnu.13038

Shao, Y., Todd, K., Shutes-David, A., Millard, S. P., Brown, K., Thomas, A., et al. (2023). Identifying probable dementia in undiagnosed black and white Americans using machine learning in veterans health administration electronic health records. *Big Data and Cognitive Computing* 7. doi:10.3390/bdcc7040167

Soysal, P., Tan, S. G., Rogowska, M., Jawad, S., Smith, L., Veronese, N., et al. (2022). Weight loss in Alzheimer's disease, vascular dementia and dementia with lewy bodies: Impact on mortality and hospitalization by dementia subtype. *International Journal of Geriatric Psychiatry* 37. doi:https://doi.org/10.1002/gps.5659

Wang, L., Zhang, Y., Chignell, M., Shan, B., Sheehan, K. A., Razak, F., et al. (2022). Boosting delirium identification accuracy with sentiment-based natural language processing: Mixed methods study. *JMIR Med Inform* 10, e38161. doi:10.2196/38161
